# Iridium Complexes of a Bis(*N*-pyrrolyl)boryl/Bis(phosphine) PBP Pincer Ligand

**DOI:** 10.1021/acs.inorgchem.4c03554

**Published:** 2024-12-09

**Authors:** Samuel R. Lee, Nattamai Bhuvanesh, Oleg V. Ozerov

**Affiliations:** Department of Chemistry, Texas A&M University, College Station, Texas 77842, United States

## Abstract

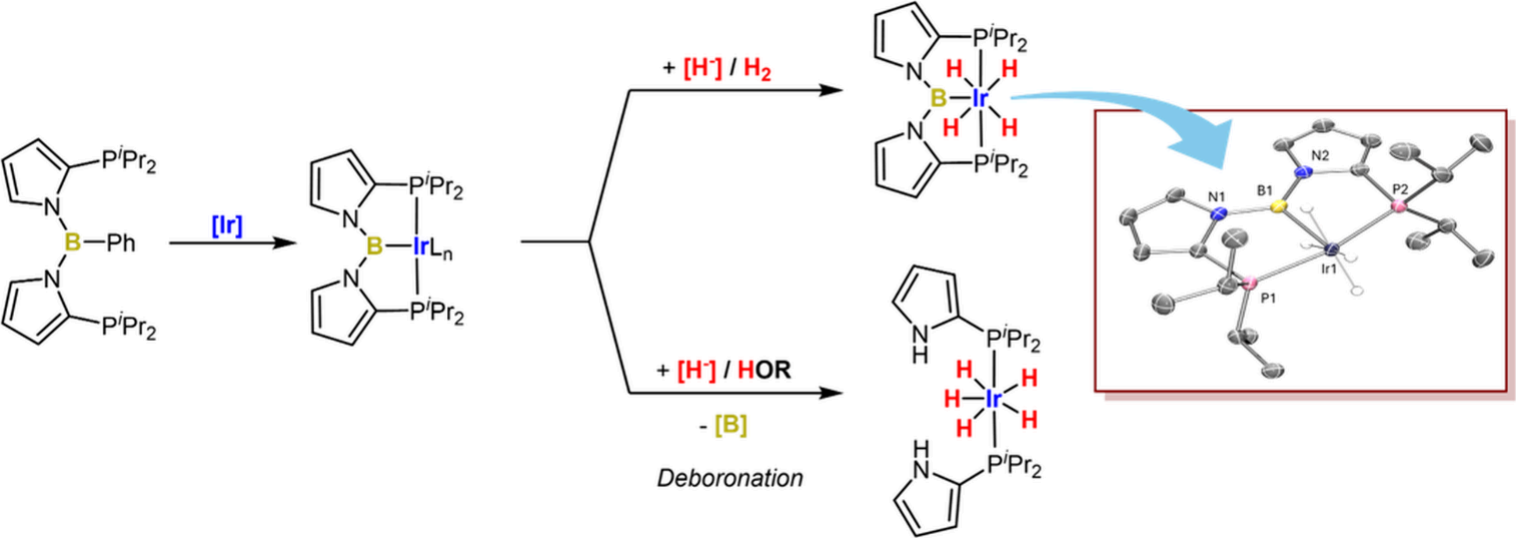

This work reports the synthesis of a bis(pyrrolylphosphino)phenyl borane (PBP)Ph (**2**) and its incorporation of Ir by metal insertion into B–Ph to afford the dipyrrolylboryl/bis(phosphine) pincer complex (PBP)Ir(Ph)Cl (**3**). Hydrogenolysis of **3** afforded (PBP)Ir(H)Cl (**4**). Compound **4** was converted into (PBP)IrCl_2_ (**5a**) via reaction with *N*-chlorosuccinimide, and exposure of **5a** to CO produced (PBP)IrCl_2_(CO) (**6a**). Compounds **5a** and **6a** were converted into their analogs (PBP)IrI_2_ (**5b**) and (PBP)IrI_2_(CO) (**6b**) via metathesis with Me_3_SiI, respectively. Treatment of either **3** or **4** with Li[HAl(O^t^Bu)_3_] under H_2_ resulted in the formation of (PBP)IrH_4_ (**7**), with traces of **4** as a persistent impurity. Attempts to access **7** via the reaction of **4** with NaBH_4_ in isopropanol led to the loss of boron from the pincer and isolation of L_2_IrH_5_ (**8**, L = 2-diisopropylphosphinopyrrole). Compounds **4**, **7**, and **8** were examined as catalysts for alkane transfer dehydrogenation but displayed only the modest activity. Solid-state structures of **6b** and **7** were established by X-ray crystallography.

## Abstract 2

This work reports rational synthetic access to the Ir complexes of a bis(*N*-pyrrolyl)boryl/bis(phosphine) PBP pincer. This ligand scaffold demonstrates a preference for boryl-iridium complexes, enabling access of a high valent boryl-iridium polyhydride. Additionally, the sensitivity of the B−N connectivity to protolysis was examined.

## Introduction

**Figure 1:**
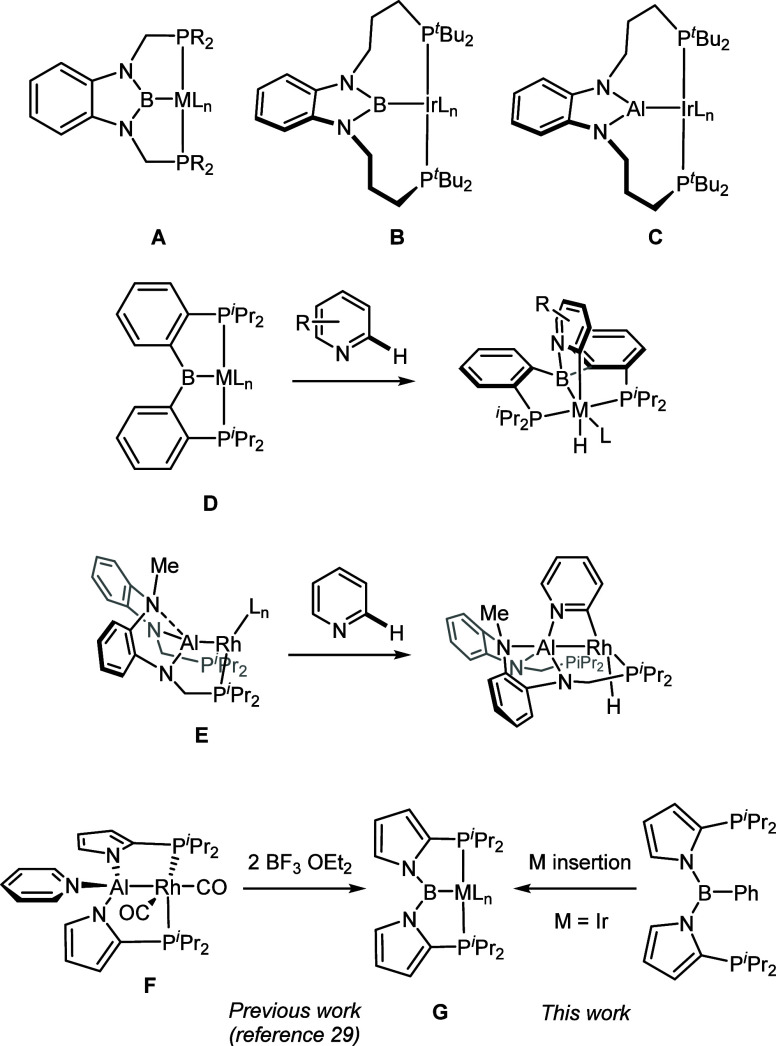
Selected structures featuring boryl-type PBP and related PAlP transition metal complexes.

Pincer complexes^1−4^ composed of a central boryl donor and two flanking phosphines have attracted increased attention for the last 15 years. The boryl moiety is among the most σ-donating and most *trans*-influencing^5,6^ X-type^7^ ligands that could be envisaged for the central pincer lynchpin. The first boryl PBP complex prepared by Yamashita at al. was of type **A** (Figure 1).^8^ The type **A** PBP ligand contains a diaminoboryl central moiety, and it has been used for a number of other transition metals.^9,10^ Yamashita et al. have additionally reported longer-tethered iterations of **A** with a diaminoboryl center **B**(11) and diaminoaluminyl center **C**(12) and their complexes of Ir. Boryl-centered pincer complexes based on the *m*-carborane cage have been reported as well.^13−15^ Our group pursued the chemistry of PBP complexes of Rh and Ir of type **D**,^16,17^ in which 1,2-phenylene connects the boron and phosphorus donor sites, while Tauchert et al. reported Pd complexes.^18^ We have been particularly interested in the selective C–H activation of pyridines and other azines by these (PBP)Rh/Ir complexes, arising from boron/metal cooperation.^19−21^ Similar selectivity has been pursued by Nakao’s group using Rh complexes supported by the aluminyl-centered PAlP ligand of type **E**.^22−25^ We recently became interested in using the 1,2-pyrrolediyl building block^26−28^ in place of 1,2-phenylene in ligands such as **D** and reported PAlP complexes of the type **F**.^29^ In the course of attempting to access a three-coordinate aluminyl in **F**-type systems, we serendipitously observed the formation of a Rh complex of a new PBP ligand (**G**) with the central bis(*N*-pyrrolyl)boryl unit (Figure 1). However, we desired more intentional pathways to the PBP complexes of type **G** and report the synthesis and characterization of these Ir complexes here.^30^

## Results and Discussion

### Synthesis and Characterization of Ir Complexes

**Scheme 1:**
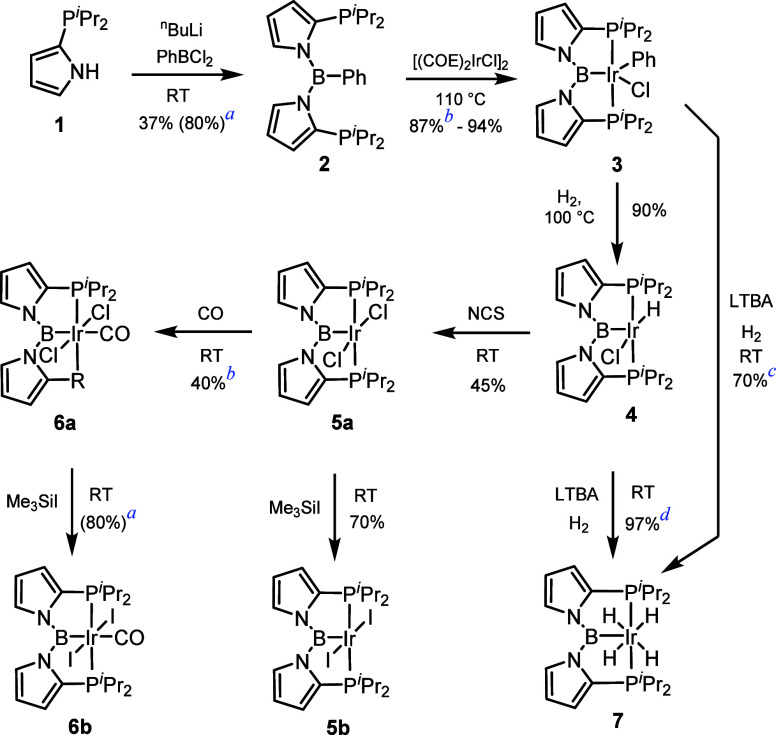
Synthesis of (PBP)^Ph^ Ligand **2** and Its Ir Complexes **3**–**7** *In situ* yield, determined by ^31^P NMR integration. Yield over two steps, precursor generated *in situ* without purification. Isolated with 90% purity. Isolated with 98% purity.

Treatment of a toluene solution of **1** with *^n^*BuLi followed by addition of PhBCl_2_ at ambient temperature rapidly afforded **2** as a crude oil, (80% purity by ^31^P{^1^H} NMR analysis), from which a 37% yield of pure material was obtained by recrystallization (Scheme 1). Mimicking the previously reported successful synthesis with the **D-Ir** species,^16^ we effected the synthesis of **3** via thermolysis of **2** with [(COE)_2_IrCl]_2_ at 110 °C for 5 h. We found that the use of crude (∼80% pure) **2** is more economical in terms of the overall transformation from **1** to **3** (see the Supporting Information).

Treatment of **3** with 1 atm of H_2_ at 100 °C for 2 h in PhF solution resulted in the formation of hydridochloride **4**, isolated in 90% yield. The hydride in **4** was readily converted into chloride by treatment of a toluene solution of **4** with *N*-chlorosuccinimide (NCS) at ambient temperature, giving **5a** in a 45% isolated yield upon workup. Compound **5a** (generated in situ) was reacted with CO upon mixing in solution, providing access to **6a** in a moderate isolated yield. Compounds **5a** and **6a** were independently treated with 2.5 equiv of Me_3_SiI in C_6_D_6_ solution at ambient temperature, and the Cl/I metathesis was monitored by NMR spectroscopy. The conversion of **5a** to **5b** was complete after 80 min. The analogous conversion of **6a** to **6b** was only about 80% complete after 24 h, but the solution eventually deposited single crystals of **6b** suitable for X-ray diffractometry studies (*vide infra*).

Synthesis of the (PBP)IrH_4_ complex **7** was approached from both **3** and **4**, treating THF solutions of the selected (P^N^B^N^P)Ir species with 1 equiv of Li[HAl(O^*t*^Bu)_3_] (LTBA) under 1 atm of H_2_ to generate **7** quantitatively *in situ*. To our surprise, workup inevitably generated an impurity of **4** regardless of the starting complex, resulting in 90% pure **7** when starting from **3** (70% yield) or 98% pure **7** when starting from **4** (97% yield). We hypothesize that the interaction between **7**, silica gel, and chlorides of Li or Al can produce a small amount of **4** during workup. Nonetheless, single crystals of **7** were grown by slow evaporation of pentane from a 98% pure material.

**Scheme 2:**
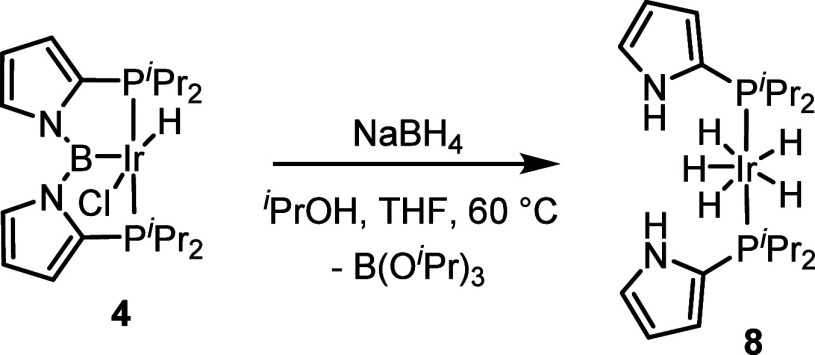
Boron Loss from the Pincer Complex **4**

Exploring other routes to **7**, we unexpectedly came across a reaction that resulted in the loss of boron from the pincer structure (Scheme 2). Subjecting **4** to thermolysis (60 °C) with excess NaBH_4_ in the ^*i*^PrOH/THF (Scheme 2) gave rise to B(O^*i*^Pr)_3_ as the only boron-containing product detectable *in situ* by ^11^B NMR spectroscopy. Upon workup, pentahydride **8** was isolated in 78% yield. Compound **8** displayed a single upfield resonance integrating to 5H. Both the chemical shift (δ −10.57 ppm) and the ^2^*J*_H–P_ value (12 Hz) align closely with the analogous (R_3_P)_2_IrH_5_ compounds in the literature,^31^ and a large *T*_1_ value (1330 ± 14 ms) is consistent with the pentahydride configuration.^32^ Interestingly, the ^1^H NMR signal for the NH protons in **8** (δ 9.47 ppm) appears downfield from that in free **1** (δ 7.45 ppm), which may suggest the presence of some dihydrogen bonding.^33^ The *T*_1_ value for this signal (1380 ± 56 ms) was found to be much smaller than for free **1** (5.6 s) and essentially the same as that for Ir–H, which has been suggested to be indicative of slow exchange by Clot.^34^ The formation of **8** suggests that alcoholysis and/or hydrogenolysis of the N–B and B–Ir bonds in **4**, or **7**, or any of the intermediates is possible. Observation of the deboronation highlights the potential downside to the use of polar and relatively more labile main group element-nitrogen bonds in the construction of multidentate ligands.^35,36^

Compounds **2** and **5**–**7** demonstrated time-averaged C_2v_ symmetry in their NMR spectra at ambient temperature, whereas **3** and **4** showed *C*_s_ symmetry as expected. All of **2**–**7** display a single ^11^B and single ^31^P NMR resonance; these data are summarized in Table 1. The observed ^11^B NMR chemical shifts are consistent with an sp^2^-hybridized boron carrying two nitrogenous substituents;^8^ however, there is notable variation in the 20–50 ppm range. The origin of this variation is difficult to pinpoint. For comparison, the Ir complexes supported by ligand type **D** and analogous to **3**, **4**, and **5a** possess ^11^B NMR chemical shifts in a much narrower range of a few ppm. The ^1^H NMR spectrum of compound **3** exhibits broadened signals for the Ir-bound C_6_H_5_ group, accompanied by shielding of one of the four CH_3_ resonances (δ 0.46 ppm). This reflects the slowed rotation about the Ir–C_6_H_5_ bond and influence of its ring current on a pair of the isopropyl methyls as discussed for similarly structured compounds elsewhere.^16^ The Ir–H resonance in **4** (δ −23.42, ^*2*^*J*_*HP*_ = 10.8 Hz) is sharp and within 1.5 ppm of that in ^**D**^**4** and is likely indicative of a similar Y-shaped geometry with no B–H interaction.^16,37^

**Table 1:** ^11^B and ^31^P NMR Chemical Shifts (in ppm) for Compounds **2**–**7**

nucleus	**2**	**3**	**4**	**5a**	**5b**	**6a**	**6b**	**7**
^11^B	40.9	39.7	38.0	27.6	22.3	51.4	50.7	51.6
^31^P	–13.9	26.8	39.7	19.3	23.4	6.0	–8.0	30.4

**Scheme 3:**
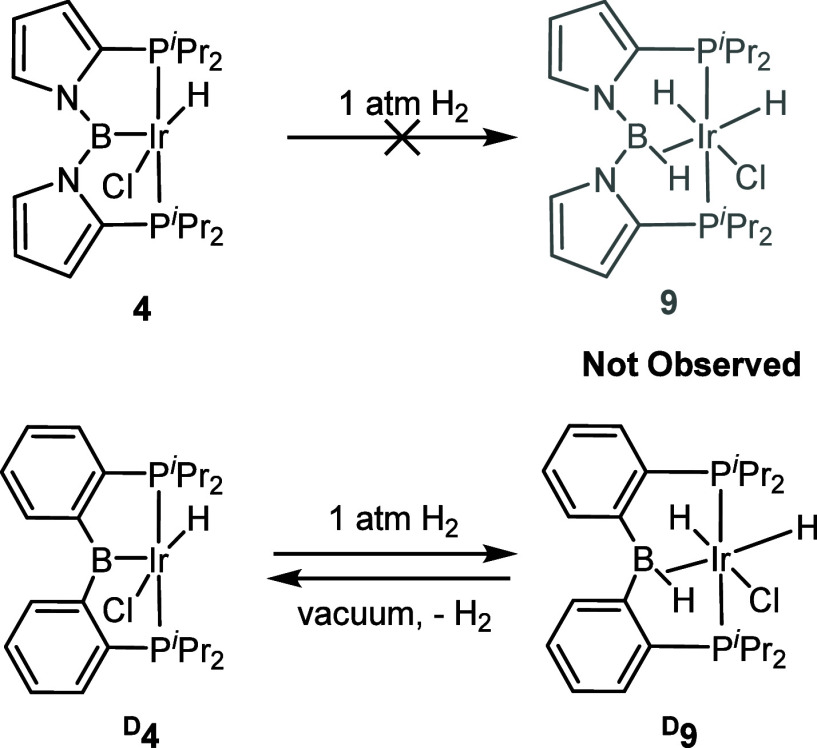
Comparison of Reactions of **4** and ^**D**^**4** with H_2_

The facile isolation of **4** from a reaction under H_2_ alerted us to the difference with the analogous chemistry with the ligand type **D**. We previously found that ^**D**^**4** under a H_2_ atmosphere reversibly added H_2_ to produce ^**D**^**9**, with a 3c–2e bond between B, H, and Ir (Scheme 3).^16,38^ However, exposure of **4** to 1 atm of H_2_, even after thermolysis (toluene-*d*_8_, 3 h, 150 °C oil bath), led to no changes in the ^1^H and ^31^P{^1^H} NMR spectra apart from the apparent partial deuteration of the Ir–H position.

**Figure 2:**
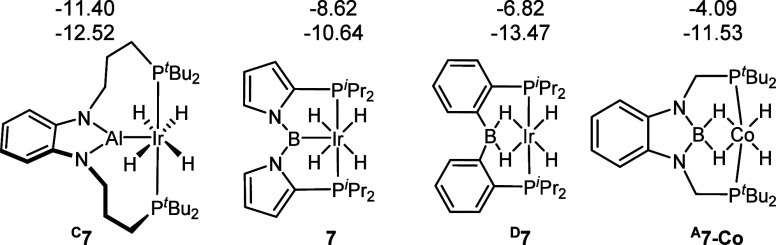
Selected (Pincer)MH_4_ complexes and the ^1^H NMR chemical shifts of their hydrides, in ppm.

**Figure 3:**
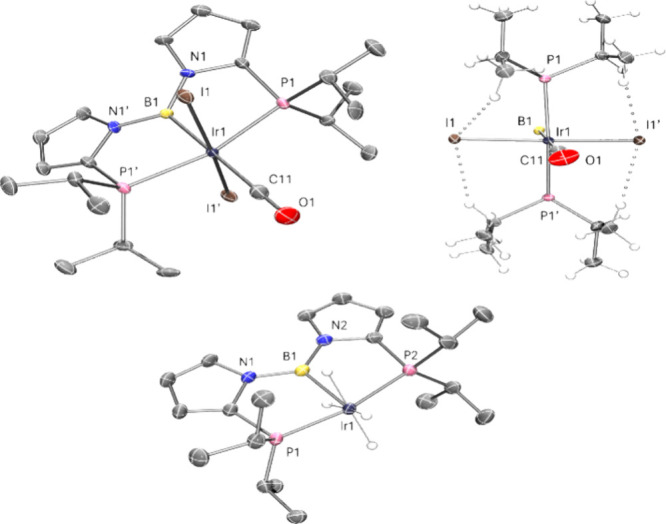
POV-ray renditions of the ORTEP drawing (50% thermal ellipsoids) of **6b** (top) and **7** (bottom), showing selected atom labeling. Solvent molecules excluded. Z′ for **6b** = 0.5. Only one of the two independent molecules of **7** in the unit cell is shown. Hydrides of **7** were placed arbitrarily to satisfy the molecular formula. Selected bond distances (Å) and angles (deg) for **6b**: Ir1–B1, 2.067(10); Ir1–P1, 2.3755(14); Ir1–I1, 2.6966(4); Ir1–I1′, 2.6967(4); Ir1–C11, 1.972(11); B1–N1, 1.465(7); P1–Ir1–P1′, 162.26(8); P1–Ir1–I1, 86.06(4); P1–Ir1–I1′, 92.79(4); C11–Ir1–B1, 180.0; I1–Ir1–I1′, 172.54(2); N1–B1–N1′, 119.2(8). Selected bond distances (Å) and angles (deg) for the molecule of **7**: Ir1–B1, 2.078(2); Ir1–P1, 2.2842(5); Ir1–P2, 2.2912(5); B1–N1, 1.481(3); B1–N2, 1.487(3); P1–Ir1–P2, 168.41(2); B1–Ir1–P1, 84.19(7); B1–Ir1–P2, 84.44(7); N1–B1–Ir1, 118.52(15); N2–B1–Ir1, 118.10(15); N1–B1–N2, 123.32(18).

Compound **7** exhibited two distinct, broadened Ir–H signals (δ −8.62 and −10.24), similarly to ^**D**^**7** (Figure 2). However, the two resonances in ^**D**^**7** possess greater disparity in chemical shifts (δ −6.82 and −13.47 ppm).^17^ We noted that in the case of Yamashita’s (PAlP)IrH_4_ (^**C**^**7**, δ −11.40 and −12.52 ppm), the difference was small,^12^ but in ^**A**^**7-Co** reported by the Peters group, it was larger (δ −4.09 and −11.53 ppm), albeit at −90 °C and under 1 atm of H_2_.^39^ The related ^**C**^**7** was interpreted by the authors as an aluminyl/tetrahydride, whereas ^**A**^**7-Co** was deemed to possess, like ^**D**^**7**, two hydride bridges between B and the transition metal and two terminal hydrides. From this perspective, it is interesting to note that the solid-state structure of **7** evinced a rather short B–Ir bond of ca. 2.08 Å, essentially indistinguishable from that in **6b**, which clearly must possess a three-coordinate boron without additional interactions (Figure 3). In contrast, ^**D**^**7** possesses an Ir–B distance of ca. 2.16 Å,^17^ which is not only 0.08 Å longer than **7** but is also ca. 0.15 Å longer than the “control” Ir–B_boryl_ distances in ^**D**^**3** and ^**D**^**4** (analogs of **3** and **4** supported by ligand of the **D** type, respectively).^16^ We would like to propose that compounds such as **7**, ^**D**^**7**, ^**C**^**7**, and ^**A**^**7-Co** populate a continuum of structures with a varying amount of B–H or Al–H interactions. It appears that **7** lies closer to the boryl/tetrahydride end of this continuum and ^**D**^**7** is closer to the dihydroborate/dihydride end. In accord with this hypothesis, there was no significant difference in the width of the hydride signals of **7** between in the ^1^H and ^1^H{^11^B} NMR spectra or of the boron resonance of **7** in the ^11^B vs ^11^B{^1^H} NMR spectra. In addition, no correlation between the hydride signals and boron resonance was evident in the ^1^H–^11^B HMQC NMR spectrum of **7**. The measured relaxation times of the ^1^H hydride resonances are also consistent with this notion (δ −8.62: *T*_1_ = 884 ± 15 ms; δ −10.24: *T*_1_ = 875 ± 12 ms).

An X-ray diffraction study of **6b** (Figure 3) confirmed the structure expected from the NMR studies, with the B–Ir–CO unit lying on a crystallographic 2-fold axis of symmetry, and thus two *trans*-iodides. The coordination environment about the Ir center is distorted octahedral, with the deviation largely coming from the chelate constraint enforcing a P–Ir–P angle of ca. 162°. The Goldman group recently reported *trans*-(PCP)IrCl_2_(CO)^40^ and discussed the importance of CH^*i*Pr^···Cl–Ir hydrogen bonding interactions for stabilizing the *trans-*Cl_2_ isomer.^41^ Some close CH^*i*Pr^···I–Ir contacts are evident in the structure of **6b**, but we did not analyze this feature in detail.

### Catalytic Alkane Transfer Dehydrogenation

Iridium complexes supported by anionic PXP pincer ligands have often been used as alkane transfer hydrogenation catalysts.^42−45^ Compounds **4** and **7** were therefore investigated as candidates for transfer dehydrogenation catalysis (Table 2), with cyclooctane (COA) as the model substrate. Compounds **4** and **7** achieved only the modest turnover numbers (TON) in reactions with either 1-hexene or *t*-butylethylene (TBE) as an acceptor. These numbers are somewhat lower than those we reported for ^**D**^**7** as a catalyst.^17^ We also tested complex **8**, whose activity was higher compared to **4** and **7**, similar to the structurally related [(^*i*^Pr_2_)_3_P]_2_IrH_5_,^46^ but still modest. The lower activities observed with 1-hexene may be attributed to the undesired alkene isomerization operant under these conditions.

**Table 2:**
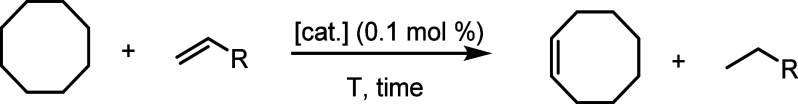
Catalytic Transfer Dehydrogenation of COA to COE Using Ir Complexes

entry	cat.	acceptor	*T* (°C)	time (h)	TON
1	**4**	1-hexene	200	24	8
2	**7**	1-hexene	200	24	15.5
3	**8**	1-hexene	200	24	60.5
4	none	1-hexene	200	24	a
5	**4**	TBE	200	24	23
6	**7**	TBE	200	24	44
7	**8**	TBE	200	24	160.5
8	none	TBE	200	24	a

a No COE was observed in the absence of the catalyst.

## Conclusions

In summary, we have been able to prepare a series of Ir complexes supported by a boryl/bis(phosphine) PBP pincer ligand with 1,2-pyrrolediyl linkers. These complexes display general similarities in the structure and reactivity to those supported by the analogous PBP ligand with 1,2-phenylene linkers (**D**) connecting B and P sites, including the modest reactivity in the catalysis of alkane transfer hydrogenation. However, the complexes of the new pyrrolic PBP ligand appear to display a greater preference for maintaining a 2-center-2-electron B–Ir bond and sp^2^-hybridized boron. This is exemplified in the absence or lesser prominence of additional nonclassical B–H/Ir interactions.

## Experimental Section

### General Considerations

Unless otherwise specified, all manipulations were performed either inside an argon-filled glovebox or by using Schlenk techniques. Pentane, toluene, and tetrahydrofuran (THF) were dried using a PureSolv MD-5 Solvent Purification System and were stored over 4 Å molecular sieves in an argon-filled glovebox. Benzene (PhH), benzene-*d*_6_ (C_6_D_6_), toluene-*d*_8_, and fluorobenzene (PhF) were dried over CaH_2_ and stored in an argon-filled glovebox over 4 Å molecular sieves prior to use. Phenylboron dichloride (PhBCl_2_) was distilled under a vacuum prior to use. Iridium precursor [(COE)IrCl]_2_^47^ and ligand **1**(26) were synthesized according to literature precedent. All other chemicals were used as received from commercial vendors. Argon was used from standard gas cylinders with 99.998% purity. All NMR spectra were acquired on Bruker Avance Neo 400 (^1^H NMR, 400.20 MHz; ^13^C NMR, 100.63 MHz; ^31^P NMR, 161.95 MHz; ^11^B NMR, 128.40), Avance Neo 500 (^1^H NMR, 500.13 MHz; ^13^C NMR, 125.77 MHz; ^31^P NMR, 202.45 MHz), Varian Inova 500 (^1^H NMR, 499.703 MHz; ^13^C NMR, 125.697 MHz; ^31^P NMR, 202.265 MHz), and Varian VnmrS 500 (^1^H NMR, 499.83 MHz; ^11^B NMR, 160.37) in denoted solvents. All chemical shifts are reported in δ (ppm). All ^1^H and ^13^C NMR spectra were referenced internally to the residual solvent signal (C_6_D_6_ at δ 7.16 for ^1^H and δ 128.06 for ^13^C NMR). ^11^B{^1^H} NMR spectra were referenced externally using neat BF_3_OEt_2_ at δ 0, and ^31^P NMR spectra were externally referenced to an 85% phosphoric acid solution δ 0. Elemental analyses were performed by Robertson Microlit Laboratories (Ledgwood, NJ). **Caution!***Multiple procedures in the experimental section involve heating a sealed vessel under a H*_*2*_*pressure. While we have performed these procedures multiple times without incident, precautions should be taken to prevent incident (proper PPE, including blast shield).*

#### Synthesis of PB^Ph^P (**2**)

To a screw-cap culture tube, 733 mg (4.0 mmol) of **1** was dissolved in 10 mL of toluene, followed by addition of 1.6 mL of ^*n*^BuLi (4.0 mmol, 2.5 M in hexanes) via a syringe and stirred for 10 min at room temperature. [***Caution!***^*n*^*BuLi is extremely pyrophoric. It must be handled using proper needle and syringe techniques.*] To this solution, 260 μL (2.0 mmol) of PhBCl_2_ was delivered by a syringe to immediately afford a yellow solution and formation of precipitate, which was stirred for 30 min at room temperature. The mixture was filtered through a short pad of Celite, and volatiles were evaporated to afford an orange oil. From a concentrated toluene solution stored overnight at −35 °C, 332 mg of colorless, fine crystals were collected via a fritted filter after washing with cold pentane (37%). ^1^H NMR (500 MHz, C_6_D_6_) δ 7.53 (d, *J* = 6.8 Hz, 2H, Pyrrole*H*), 7.24 (t, *J* = 7.4 Hz, 1H, Ph*H*), 7.14 (t, *J* = 7.6 Hz, 2H, Ph*H*), 6.93 (br s, 2H, Ph*H*), 6.69 (br s, 2H, Pyrrole*H*), 6.42 (br s, 2H, Pyrrole*H*), 1.84 (hept, *J* = 7.0 Hz, 4H, C*H*Me_2_), 1.00 (dd, *J* = 12.1, 7.0 Hz, 12H, CH*Me*_*2*_), 0.97 (dd, *J* = 13.9, 7.0 Hz, 12H, CH*Me*_*2*_). ^31^P{^1^H} NMR (202 MHz, C_6_D_6_) δ −13.9. ^11^B{^1^H} NMR (128 MHz, C_6_D_6_) δ 40.9. ^13^C{^1^H} NMR (126 MHz, C_6_D_6_) δ 137.3 (br, Ph*C*), 135.8 (d, *J*_*P–C*_ = 18.9 Hz, Pyrrole*C*), 131.7–131.5 (m, overlapping signals, Pyrrole*C* + Ph*C*), 127.8 (Ph*C*), 122.5 (d, *J*_*P–C*_ = 5.0 Hz, Pyrrole*C*), 112.8 (Pyrrole*C)*, 25.6–25.1 (m, overlapping signals, *C*HMe_2_), 20.5–19.9 (m, overlapping signals, CH*Me*_*2*_). HRMS (ESI) for C_26_H_40_BN_2_P_2_^+^ (**[2-H]**^**+**^) Calc: 453.27, Found: 453.27.

#### Synthesis of (PBP)Ir(Ph)(Cl) (**3**)

##### Method A, from Purified **2**

To a screw-cap culture tube charged with 224 mg (0.25 mmol) of [(COE)_2_IrCl]_2_ and a stir bar, 226 mg (0.50 mmol) of **2** was added and dissolved in 5 mL of toluene before heating in a 110 °C oil bath for 5 h. The toluene solution was filtered through a small pad of silica gel. Removal of volatiles gave 318 mg of **3** as amber crystals (94%).

##### Method B, Sequential Synthesis without Purification of **2**

To a screw-cap culture tube, 275 mg (1.5 mmol) of **1** was loaded with a stir bar in 5 mL of toluene before addition of 0.6 mL (1.5 mmol, 2.5 M in hexanes) of ^*n*^BuLi via syringe at room temperature. This solution was stirred for 10 min before 97.5 μL (0.75 mmol) of PhBCl_2_ was added and stirred for a further 30 min to give a yellow solution with precipitate. This solution was filtered through a pad of Celite, and volatiles were removed to afford crude **2** as an oil (0.60 mmol, 80% purity determined by ^31^P NMR, Figure S5). The oil containing **2** was dissolved in 5 mL of toluene before addition of 251 mg (0.28 mmol) of [(COE)_2_IrCl]_2_ and a stir bar. The toluene solution was heated in a 110 °C oil bath for 5 h, and the solution was filtered through a short pad of silica; volatiles were removed. The residue was washed with pentane (3 × 2 mL) and dried under vacuum to give 332 mg of **3** as a dandelion powder (87% based on Ir, 65% based on **1**). ^1^H NMR (500 MHz, C_6_D_6_) δ 7.64 (m, 2H, Pyrrole*H*), 7.12–6.74 (brs, 2H, Ph*H*), 6.61–6.56 (m, 3H, Ph*H*), 6.55 (t, *J* = 3.0 Hz, 2H, Pyrrole*H*), 6.40 (d, *J* = 3.0 Hz, 2H, Pyrrole*H*), 3.25 (hept, *J* = 6.9 Hz, 2H, C*H*Me_2_), 2.30 (m, 2H, C*H*Me_2_), 1.16 (dvt, *J*_*H–H*_ ≈ *J*_*H–P*_ = 7.4 Hz, 6H, CH*Me*_*2*_), 1.07–0.98 (overlapping signals, 12H, CH*Me*_*2*_), 0.46 (dvt, *J*_*H–H*_ ≈ *J*_*H–P*_ = 7.5 Hz, 6H, CH*Me*_*2*_). ^31^P{^1^H} NMR (202 MHz, C_6_D_6_) δ 26.8. ^11^B{^1^H} NMR (128 MHz, C_6_D_6_) δ 39.7. ^13^C{^1^H} NMR (126 MHz, C_6_D_6_) δ 135.3 (vt, *J*_*P–C*_ = 32.8 Hz, Pyrrole*C*), 127.2 (br, Ph*C*), 125.2 (vt, *J*_*P–C*_ = 5.0 Hz, Pyrrole*C*), 125.1 (vt, *J*_*P–C*_ = 6.3 Hz), 122.3 (Ph*C*), 119.0 (Pyrrole*C*), 116.5 (vt, *J*_*P–C*_ = 3.8, Pyrrole*C*), 23.9–23.4 (m, overlapping signals, *C*HMe_2_), 19.7 (d, *J*_*P–C*_ = 47.9 Hz, CH*Me*_*2*_), 18.0–17.2 (m, overlapping signals, CH*Me*_*2*_). Elem. Anal. Calcd for C_26_H_39_BClIrN_2_P_2_: C, 45.92; H, 5.78; N, 4.12. Found: C, 46.15; H, 5.93; N, 4.00. *Elemental analysis was conducted on compound***3***prepared via* Method B*.*

#### Synthesis of (PBP)IrHCl (**4**)

To a 25 mL PTFE-stoppered round-bottomed flask, 321 mg of **3** (0.47 mmol) was added and dissolved in 10 mL of PhF. The solution was degassed via three cycles of freeze–pump–thaw and refilled with 1 atm of H_2_, and the flask was placed in a 100 °C oil bath with stirring for 2 h. The solution was again degassed by freeze–pump–thaw, and volatiles were removed to reveal a yellow residue. The residue was triturated with 2 mL of pentane, and the solid was dried under vacuum to yield 256 mg of **4** as a yellow powder (90%). ^1^H NMR (500 MHz, C_6_D_6_) δ 7.37 (brs, 2H, Pyrrole*H*), 6.53 (brs, 2H, Pyrrole*H*), 6.43 (brs, 2H, Pyrrole*H*), 3.07 (m, 2H, C*H*Me_2_), 2.32 (m, 2H, C*H*Me_2_), 1.22–1.06 (overlapping signals, 18H, CH*Me*_*2*_), 0.98 (dvt, *J*_*H–H*_ ≈ *J*_*H–P*_ = 7.8 Hz, 6H, CH*Me*_*2*_), −23.42 (t, *J*_*H–P*_ = 10.8 Hz, Ir–*H*). (400 MHz, toluene-*d*_8_) δ 7.30 (brs, 2H, Pyrrole*H*), 6.50 (t, *J* = 3.0 Hz, 2H, Pyrrole*H*), 6.39 (d, *J* = 3.0 Hz, 2H, Pyrrole*H*), 3.00 (m, 2H, C*H*Me_2_), 2.29 (m, 2H, C*H*Me_2_), 1.16–1.05 (m, 18H, CH*Me*_*2*_), 0.96 (dvt, *J*_*H–H*_ ≈ *J*_*H–P*_ = 7.4 Hz, 6H, CH*Me*_*2*_), −23.46 (t, *J* = 10.9 Hz, 1H). ^31^P{^1^H} NMR (202 MHz, C_6_D_6_) δ 39.7. ^11^B{^1^H} NMR (128 MHz, C_6_D_6_) δ 38.0. ^13^C{^1^H} NMR (126 MHz, C_6_D_6_) δ 136.7 (t, *J*_*P–C*_ = 32.4 Hz, Pyrrole*C*), 124.7 (t, *J*_*P–C*_ = 4.4 Hz, Pyrrole*C*), 117.9 (brs, Pyrrole*C*), 116.2 (t, *J*_*P–C*_ = 3.1 Hz, Pyrrole*C*), 24.9 (vt *J*_*P–C*_ = 16.5 Hz, *C*HMe_2_), 22.7 (vt, *J*_*P–C*_ = 16.1 Hz, *C*HMe_2_), 20.0 (brs, CH*Me*_*2*_), 18.8 (CH*Me*_*2*_), 18.2 (vt, *J*_*P–C*_ = 3.4 Hz, CH*Me*_*2*_), 16.8 (CH*Me*_*2*_). Elem. Anal. Calcd for C_20_H_35_BClIrN_2_P_2_: C, 39.78; H, 5.84; N, 4.64. Found: C, 39.94; H, 5.70; N, 4.54.

### Thermolysis of **4** under 1 atm of H_2_

A J. Young NMR tube was loaded with 24 mg of **4** (0.04 mmol) dissolved in 0.5 mL of toluene-*d*_8_, and the solution was degassed via two cycles of freeze–pump–thaw before refill with 1 atm of H_2_. The tube was placed in a 150 °C oil bath and monitored by NMR analysis at the 1 and 3 h time points, revealing a 77% decrease in the Ir–H intensity, presumably owing to the H/D exchange.

#### Synthesis of (PBP)IrCl_2_ (**5a**)

To a 20 mL scintillation vial charged with a stir bar were added 39 mg of **4** (0.06 mmol) and 10 mg of NCS (0.08 mmol) and dissolved in 2 mL of toluene with stirring for 1 h. Volatiles were removed to afford a green residue, which was extracted in pentane, and volatiles were removed again. The bright yellow powder was suspended in benzene and passed through a plug of Celite layered on silica gel to afford a yellow solution. Freeze-drying the benzene solution afforded 17 mg of **5a** as a bright yellow powder (45%). ^1^H NMR (400 MHz, C_6_D_6_) δ 7.32 (brs, 2H, Pyrrole*H*), 6.55 (d, *J*_*H–H*_ = 3.0 Hz, 2H, Pyrrole*H*), 6.33 (t, *J* = 3.0 Hz, 2H, Pyrrole*H*), 3.13 (m, 4H, C*H*Me_2_), 1.39–1.24 (overlapping signals, 24H, CH*Me*_*2*_). ^31^P{^1^H} NMR (202 MHz, C_6_D_6_) δ 19.3. ^11^B{^1^H} NMR (160 MHz, C_6_D_6_) δ 27.6. ^13^C{^1^H} NMR (126 MHz, C_6_D_6_) δ 135.9 (vt, *J*_*P–C*_ = 32.1 Hz, Pyrrole*C*), 124.6 (Pyrrole*C*), 120.1 (Pyrrole*C*), 116.0 (Pyrrole*C*), 23.7 (t, *J*_*P–C*_ = 15.9 Hz, *C*HMe_2_), 20.1 (CH*Me*_2_), 18.7 (CH*Me*_2_).

#### *In Situ* Synthesis of (PBP)IrI_2_ (**5b**)

To a J. Young NMR tube containing 18 mg of **5a** (0.028 mmol) in 0.5 mL of C_6_D_6_, 10 μL of iodotrimethylsilane (0.07 mmol, TMSI) was added and the sealed tube was shaken vigorously. Conversion to **5b** was tracked by ^31^P{^1^H} NMR, with 89% conversion in 20 min and completion in 80 min. Volatiles were removed, and the residue was triturated with pentane to give 16 mg of **5b** (70%) in >95% purity by ^1^H NMR. ^1^H NMR (400 MHz, C_6_D_6_) δ 7.36 (m, 2H, Pyrrole*H*), 6.55 (d, *J* = 3.1 Hz, 2H, Pyrrole*H*), 6.33 (t, *J* = 3.1 Hz, 2H, Pyrrole*H*), 3.61 (m, 4H, C*H*Me_2_), 1.33 (dvt, *J*_*H–H*_ ≈ *J*_*H–P*_ = 7.6 Hz, 12H CH*Me*_*2*_) 1.22 (dvt, *J*_*H–H*_ ≈ *J*_*H–P*_ = 7.4 Hz, 12H, CH*Me*_*2*_). ^31^P{^1^H} NMR (162 MHz, C_6_D_6_) δ 23.4. ^11^B{^1^H} NMR (128 MHz, C_6_D_6_) δ 22.3. ^13^C{^1^H} NMR (106 MHz, C_6_D_6_) δ 136.0 (vt, *J*_*C–P*_ = 32.7 Hz, Pyrrole*C*), 124.8 (vt, *J*_*C–P*_ = 5.0 Hz, Pyrrole*C*), 120.4 (vt, *J*_*C–P*_ = 2.0 Hz, Pyrrole*C*), 115.7 (vt, *J*_*C–P*_ = 3.0 Hz, Pyrrole*C*), 27.1 (vt, *J*_*C–P*_ = 16.1 Hz, *C*HMe_2_), 19.9 (CH*Me*_*2*_), 19.4 (CH*Me*_*2*_).

#### Synthesis of *trans-*(PBP)IrCl_2_(CO) (**6a**)

A J. Young NMR tube was charged with 30 mg of **4** (0.05 mmol) and 10 mg of *N*-chlorosuccinimide (0.08 mmol) with 0.6 mL of benzene. The tube was placed in a 100 °C oil bath for 30 min then allowed to cool to room temperature. The tube was degassed via two cycles of freeze–pump–thaw, refilled with 1 atm CO, and shaken vigorously for 2 min. [**Caution!***Carbon monoxide (CO) is an extremely toxic gas; caution should be taken when conducting procedures requiring its handling, including proper ventilation.*] The solution was passed through a pad of silica and further eluted with benzene. Volatiles were removed under a vacuum to afford a yellow residue, which was triturated with pentane to give 13 mg of **6a** (40%) as an off-white powder. ^1^H NMR (500 MHz, C_6_D_6_) δ 7.33 (m, 2H, Pyrrole*H*), 6.58 (d, J = 3.0 Hz, 2H, Pyrrole*H*), 6.54 (t, J = 3.0 Hz, 2H, Pyrrole*H*), 3.00 (m, 4H, C*H*Me_2_), 1.37 (dvt, *J*_*H–H*_ ≈ *J*_*H–P*_ = 7.8 Hz, 12H, CH*Me*_*2*_), 1.32 (dvt, *J*_*H–H*_ ≈ *J*_*H–P*_ = 7.8 Hz, 12H, CH*Me*_*2*_). ^31^P{^1^H} NMR (202 MHz, C_6_D_6_) δ 6.0. ^11^B{^1^H} NMR (128 MHz, C_6_D_6_) δ 51.4. ^13^C{^1^H} NMR (101 MHz, C_6_D_6_) δ 177.7 (br Ir-*C*O), 139.2 (vt, *J*_*P–C*_ = 25.1 Hz, Pyrrole*C*), 125.2 (vt, *J*_*P–C*_ = 4.7 Hz, Pyrrole*C*), 121.4 (vt, *J*_*P–C*_ = 2.8 Hz, Pyrrole*C*), 118.0 (vt, *J*_*P–C*_ = 3.5 Hz, Pyrrole*C*), 23.8 (vt, *J*_*P–C*_ = 16.6 Hz, *C*HMe_2_), 20.7 (CH*Me*_2_), 18.9 (CH*Me*_2_). IR, ν_CO_ = 2040 cm^–1^.

#### Synthesis of *trans-*(PBP)IrI_2_(CO) (**6b**)

To a J. Young NMR tube containing 12 mg of **6a** (0.02 mmol) in 0.5 mL of C_6_D_6_ was added 7 μL of iodotrimethylsilane (0.05 mmol) via a microsyringe, and the tube was stirred for 24 h, giving **6b** in 80% abundance by ^31^P{^1^H} NMR analysis. Upon standing overnight, crystals were observed that were suitable for X-ray analysis. ^1^H NMR (400 MHz, C_6_D_6_) δ 7.36 (m, 2H, Pyrrole*H*), 6.60 (d, *J* = 2.9 Hz, 2H, Pyrrole*H*), 6.47 (t, *J* = 3.0 Hz, 2H, Pyrrole*H*), 3.51 (m, 4H, C*H*Me_2_), 1.39–1.25 (ovlp. m, 24H, CH*Me*_*2*_). ^31^P{^1^H} NMR (162 MHz, C_6_D_6_) δ −7.97. ^11^B{^1^H} NMR (128 MHz, C_6_D_6_) δ 50.7. IR, ν_CO_ = 2025 cm^–1^.

#### *In Situ* Observations and Synthesis of (PBP)IrH_4_ (**7**)

##### Method A

To a 25 mL of PTFE-stoppered round-bottom flask charged with a stir bar, 136 mg of **3** (0.2 mmol) was added and dissolved in 2 mL of THF. In a separate vial, 51 mg of LTBA (0.2 mmol) was dissolved in 1 mL of THF then added to the round-bottom flask, washing residual LTBA into the flask with 2 × 1 mL of THF. The flask was sealed, removed from the glovebox, allowed to stir for 5 min at room temperature before submerging in a liquid nitrogen bath, and then degassed via three cycles of freeze–pump–thaw. After degassing, the solution was warmed to room temperature, and the flask was refilled with 1 atm of H_2_, stirring continued for 4 h. The solution was then degassed via three cycles of freeze–pump–thaw, and an aliquot was taken to confirm **7** as the sole product by ^31^P{^1^H} NMR (Figure S32). Volatiles were removed under vacuum; then the yellow residue washed with pentane (3 × 2 mL). The residue was dissolved in minimal PhH and filtered through a short pad of silica gel, and volatiles were removed again under vacuum to give 80.2 mg (70%) of a bright yellow powder. The powder was characterized by multinuclear NMR, revealing a composition of 10% **4** to 90% **7**.

##### Method B

A J. Young NMR tube was charged with 24 mg of **4** (0.04 mmol) and 10 mg of LTBA (0.04 mmol) before addition of 0.6 mL of THF. The tube was agitated for 5 min and then frozen in a liquid nitrogen bath before degassing and refilling with 1 atm of H_2_ with shaking. The tube was left at room temperature for 16 h; at which time, **7** was observed in 95% purity by ^31^P{^1^H} NMR. The contents of the tube were washed into a scintillation vial with PhH, and the solution was passed through a silica pipet filter before removal of volatiles under vacuum to give a yellow residue. The residue was triturated with pentane and dried under vacuum to give 22 mg of **7** (97%, 98% pure by ^31^P{^1^H} NMR, Figure S34) as a yellow powder. Crystals suitable for X-ray crystallography were grown from the slow evaporation of a pentane solution containing **7**. ^1^H NMR (500 MHz, C_6_D_6_) δ 7.33 (m, 2H, Pyrrole*H*), 6.65 (t, *J* = 3.0 Hz, 2H, Pyrrole*H*), 6.47 (d, *J* = 3.0 Hz 2H, Pyrrole*H*), 1.94 (m, 4H, C*H*Me_2_), 1.10 (m, 12H, CH*Me*_*2*_), 0.95 (dvt, *J*_*H–H*_ ≈ *J*_*H–P*_ = 7.1 Hz, 12H, CH*Me*_*2*_), −8.62 (br s, 2H, Ir–*H*, *T*_1_ = 884 ± 15 ms), −10.24 (brs, 2H, Ir–*H*, *T*_1_ = 875 ± 12 ms). ^31^P{^1^H} NMR (202 MHz, C_6_D_6_) δ 30.4. ^11^B{^1^H} NMR (128 MHz, C_6_D_6_) δ 51.6. ^13^C{^1^H} NMR (101 MHz, C_6_D_6_) δ 140.71 (t, *J* = 31.2 Hz, Pyrrole*C*), 123.93 (t, *J* = 4.0 Hz, Pyrrole*C*), 118.36 (t, *J* = 2.5 Hz, Pyrrole*C*), 115.40 (t, *J* = 3.5 Hz, Pyrrole*C*), 25.98 (t, *J* = 18.0 Hz, *C*HMe_2_), 19.60 (t, *J* = 2.7 Hz, CH*Me*_*2*_), 18.69 (CH*Me*_*2*_).

#### Synthesis of P_2_IrH_5_ (**8**)

To a 50 mL PTFE-stoppered round-bottom flask charged with a stir bar, 150 mg of **4** (0.249 mmol) and 38 mg of NaBH_4_ (1.00 mmol) were added sequentially before dissolving the mixture in 4 mL of THF and 4 mL of ^*i*^PrOH. The reaction was stirred in a 60 °C oil bath for 1 h, and an aliquot was taken for ^11^B{^1^H} NMR analysis, revealing B(O^i^Pr)_3_ as the sole B-containing product (Figure S40). Volatiles were removed to afford a yellowish residue, which was suspended in PhH and passed through a short pad of Celite layered on silica. Volatiles were removed via lyophilization to give 110 mg of **8** as a colorless powder (78%). ^1^H NMR (400 MHz, C_6_D_6_) δ 9.47 (s, 2H, N*H*, *T*_1_ = 1380 ± 56 ms), 6.53 (brs, 2H, Pyrrole*H*), 6.49 (m, 2H, Pyrrole*H*), 6.34 (brs, 2H, Pyrrole*H*), 1.88 (m, 4H, C*H*Me_2_), 1.07 (dvt, *J*_*H–H*_ ≈ *J*_*H–P*_ = 7.2 Hz 12H, CH*Me*_*2*_), 0.96 (q, *J*_*H–H*_ ≈ *J*_*H–P*_ = 7.1 Hz, 12H, CH*Me*_*2*_), −10.57 (t, *J* = 12.3 Hz, 5H, Ir–*H*, *T*_1_ = 1330 ± 14 ms). ^31^P NMR (162 MHz, C_6_D_6_) δ 22.4. ^13^C NMR (101 MHz, C_6_D_6_) δ 120.4 (t, *J* = 3.6 Hz, Pyrrole*C*), 119.7 (t, *J* = 31.6 Hz, Pyrrole*C*), 112.5 (t, *J* = 2.7 Hz, Pyrrole*C*), 111.3 (t, *J* = 3.1 Hz, Pyrrole*C*), 28.3 (t, *J* = 19.6 Hz, *C*HMe_2_), 19.1 (t, *J* = 2.4 Hz, CH*Me*_*2*_), 18.2 (CH*Me*_*2*_).

### General Procedure for Transfer Dehydrogenation of Cyclooctane Using **4**, **7**, and **8**

A 25 mL PTFE-stoppered side arm Schlenk flask was charged with 0.01 mmol of the chosen catalyst (**4**; 6.0 mg, **7**; 5.7 mg, and **8**; 5.6 mg), 10.0 mmol of COA (1.35 mL), and 10.0 mmol of the chosen acceptor olefin (1-hexene; 1.25 mL, TBE; 1.29 mL). The flask was sealed and placed in a 200 °C oil bath for 24 h. The flask was allowed to cool to room temperature before an internal standard of 0.50 mL of mesitylene (3.59 mmol) was added, and an aliquot was dissolved in C_6_D_6_ for ^1^H NMR analysis. TON was calculated on the basis of integration of the COE olefinic resonance against the mesitylene internal standard.

## Supporting Information Available

Details of X-ray crystallographic procedures and pictorial NMR spectra (PDF)
