# Supplementary material for: Iridium Complexes of a Bis(N-pyrrolyl)boryl/Bis(phosphine) PBP Pincer Ligand
Source: Inorg Chem. 2024 Dec 9;63(51):24133–40. doi: 10.1021/acs.inorgchem.4c03554 (PMC11684021; doi:10.1021/acs.inorgchem.4c03554)
Supplement: Supplementary file 1 — ic4c03554_si_001.pdf [file ic4c03554_si_001.pdf]

Supporting Information

Iridium Complexes of a Bis(N-pyrrolyl)boryl/Bis(phosphine) PBP Pincer Ligand

*Samuel R. Lee, Nattamai Bhuvanesh, and Oleg V. Ozerov**

Department of Chemistry, Texas A&M University, 3255 TAMU, College Station, TX 77842, USA.

ozerov@chem.tamu.edu

Contents

I. Selected NMR Data Tables 3

II. NMR Spectra 4

III. X-Ray Structural Analysis 47

IV. SI References 50

I. Selected NMR Data Tables

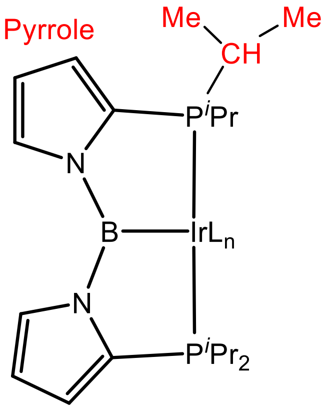

Table S1. Chemical Shifts (in ppm) of ^{1}H NMR Spectra for Pyrrolyl Signals for **1-8**.

Compound	Pyrrole*H*	C*H*	*Me*$_2$
**1**	6.52-6.49, 6.37	1.84	1.01, 0.95
**2**	7.53, 6.62, 6.42	1.84	1.00, 0.97
**3**	7.64, 6.55, 6.40	3.25, 2.3	1.16, 1.07-0.98, 0.46
**4**	7.37, 6.53, 6.43	3.07, 2.32	1.22-1.06, 0.98
**5a**	7.32, 6.55, 6.33	3.13	1.39-1.24
**5b**	7.36, 6.55, 6.33	3.61	1.33, 1.22
**6a**	7.33, 6.58, 6.54	3.00	1.39-1.30
**6b**	7.36, 6.60, 6.47	3.51	1.39-1.25
**7**	7.33, 6.65, 6.47	1.94	1.11, 0.95
**8**	6.53, 6.49, 6.34	1.88	1.07, 0.96

Table S2. Chemical Shifts (in ppm) of ^{13}C{^{1}H} NMR Spectra for Pyrrolyl Signals for **1-8**.

Compound	Pyrrole*C*	*C*H	*Me*
**1**[a]	124.3, 121.5, 117.6, 110.3	24.4	20.8, 19.7
**2**	135.8, 131.7-131.5,[b] 122.5, 112.8	25.6-25.1	20.5-19.9
**3**	135.3, 125.2, 119.0, 116.5	23.9-23.4	19.7, 18.0-17.2
**4**	136.7, 124.7, 117.9, 116.2	24.9, 22.7	20.0, 18.8, 18.2, 16.8
**5a**	135.9, 124.6, 120.1, 116.0	23.7	20.1, 18.7
**5b**	136.0, 124.8, 120.4, 115.7	27.1	19.9, 19.4
**6a**	139.2, 125.2, 121.4, 118.0	23.8	20.7, 18.9
**7**	140.7, 123.9, 118.4, 115.4	26.0	19.6, 18.7
**8**	120.4, 119.7, 112.5, 111.3	28.3	19.1, 18.2

[a]From SI reference 3.

II. NMR Spectra

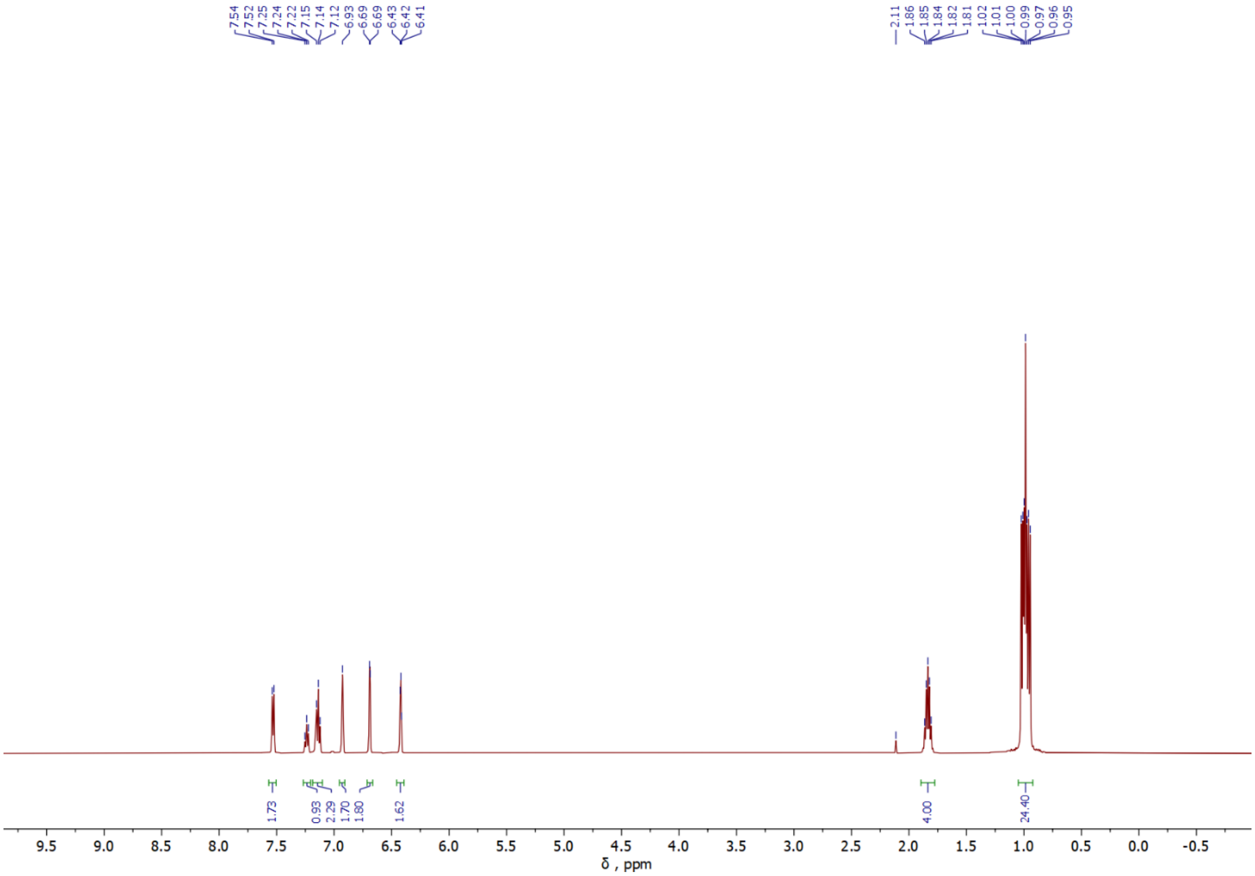

Figure S1. ^{1}H NMR (500 MHz, C_6D_6) spectrum of **2**.

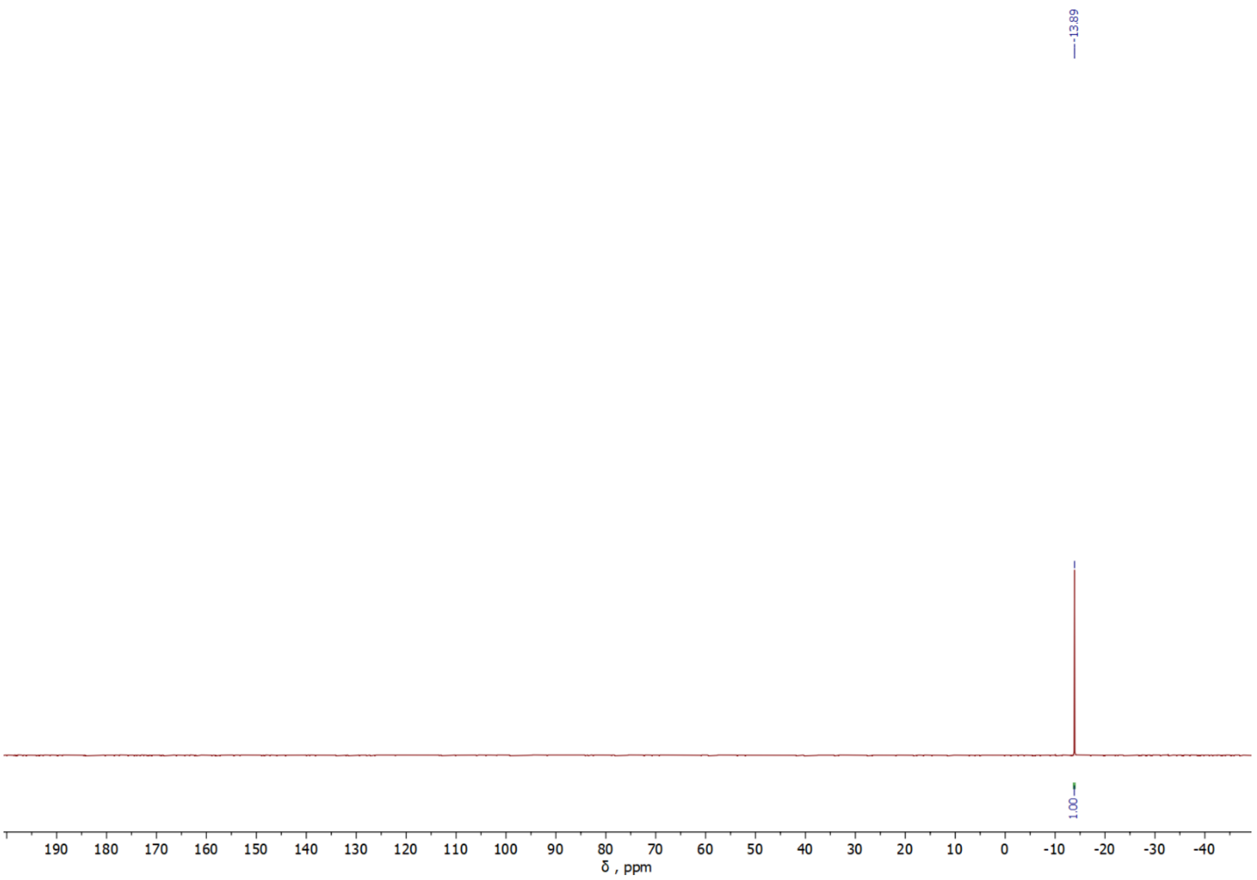

Figure S2. $^{31}P\{^1H\}$ (202 MHz, C_6D_6) spectrum of **2**.

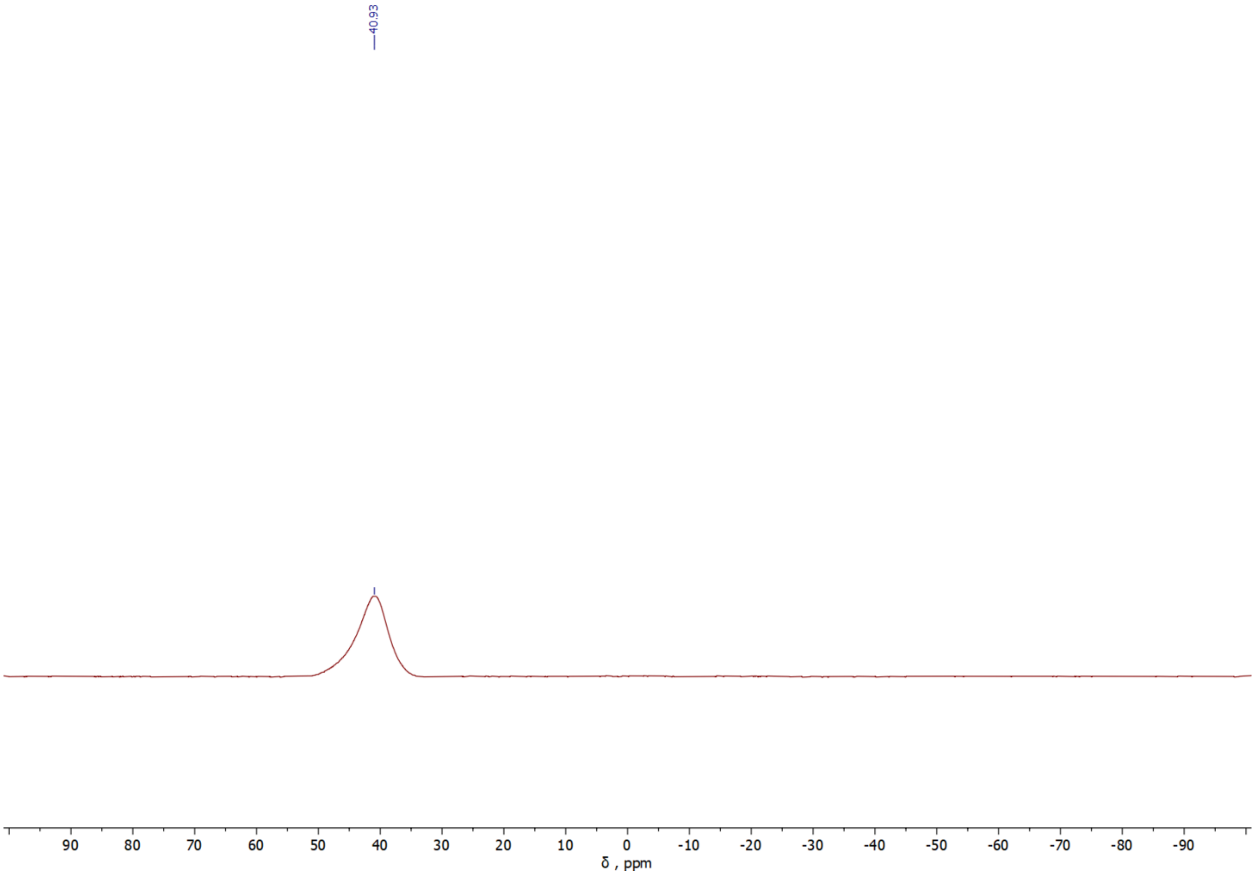

Figure S3. $^{11}B\{^{1}H\}$ NMR (128 MHz, C_6D_6) spectrum of **2**.

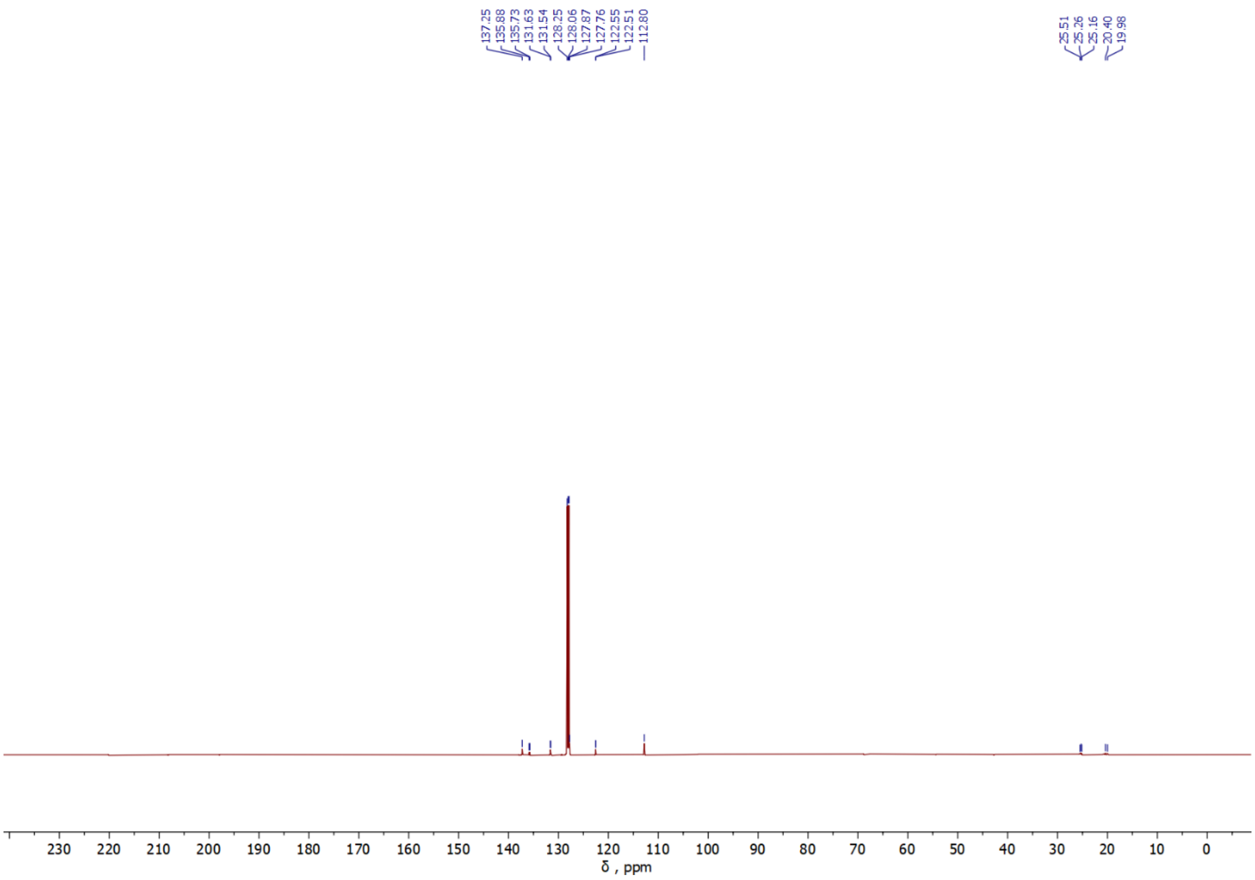

Figure S4. $^{13}C\{^{1}H\}$ NMR (126 MHz, C_6D_6) spectrum of **2**.

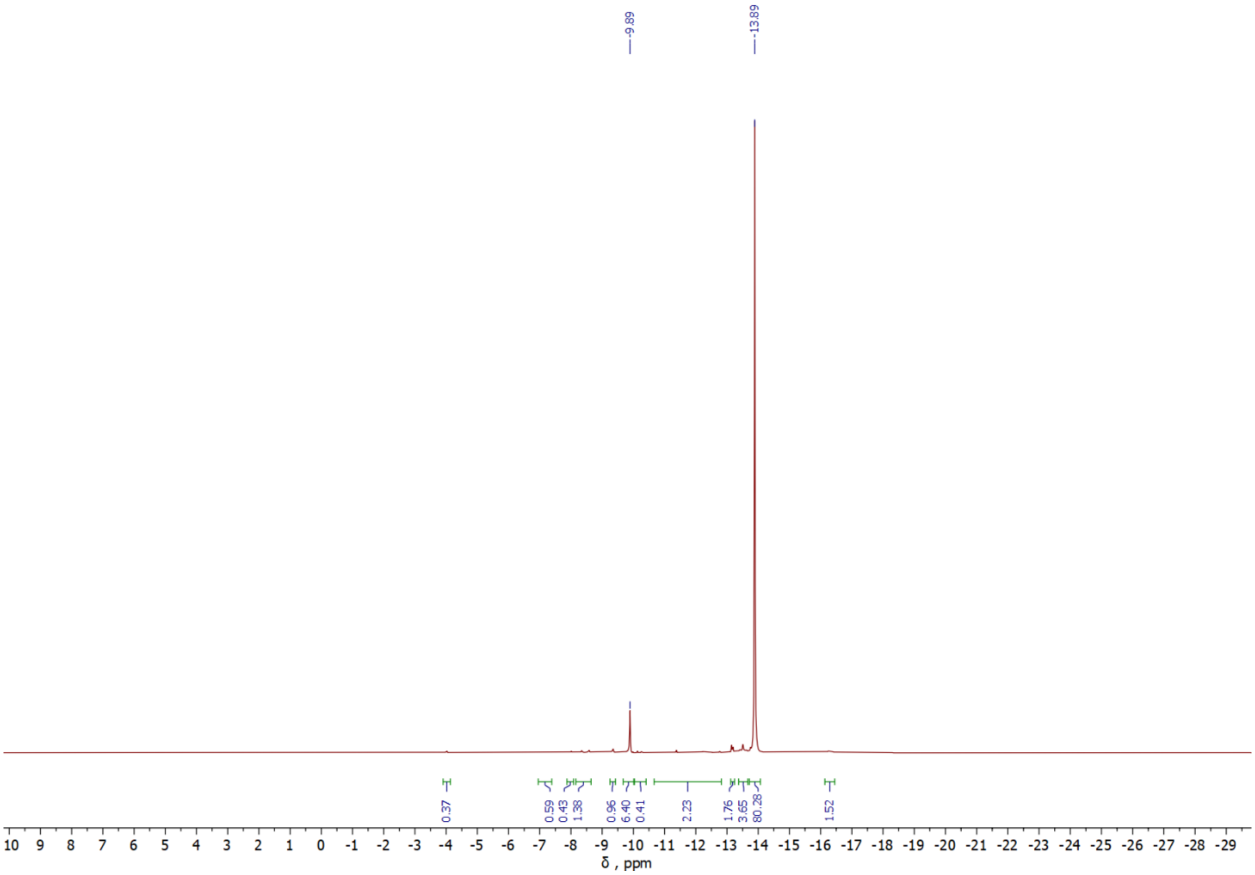

Figure S5. $^{31}P\{^{1}H\}$ (202 MHz, C_6D_6) spectrum of crude **2** prepared in **Method B**.

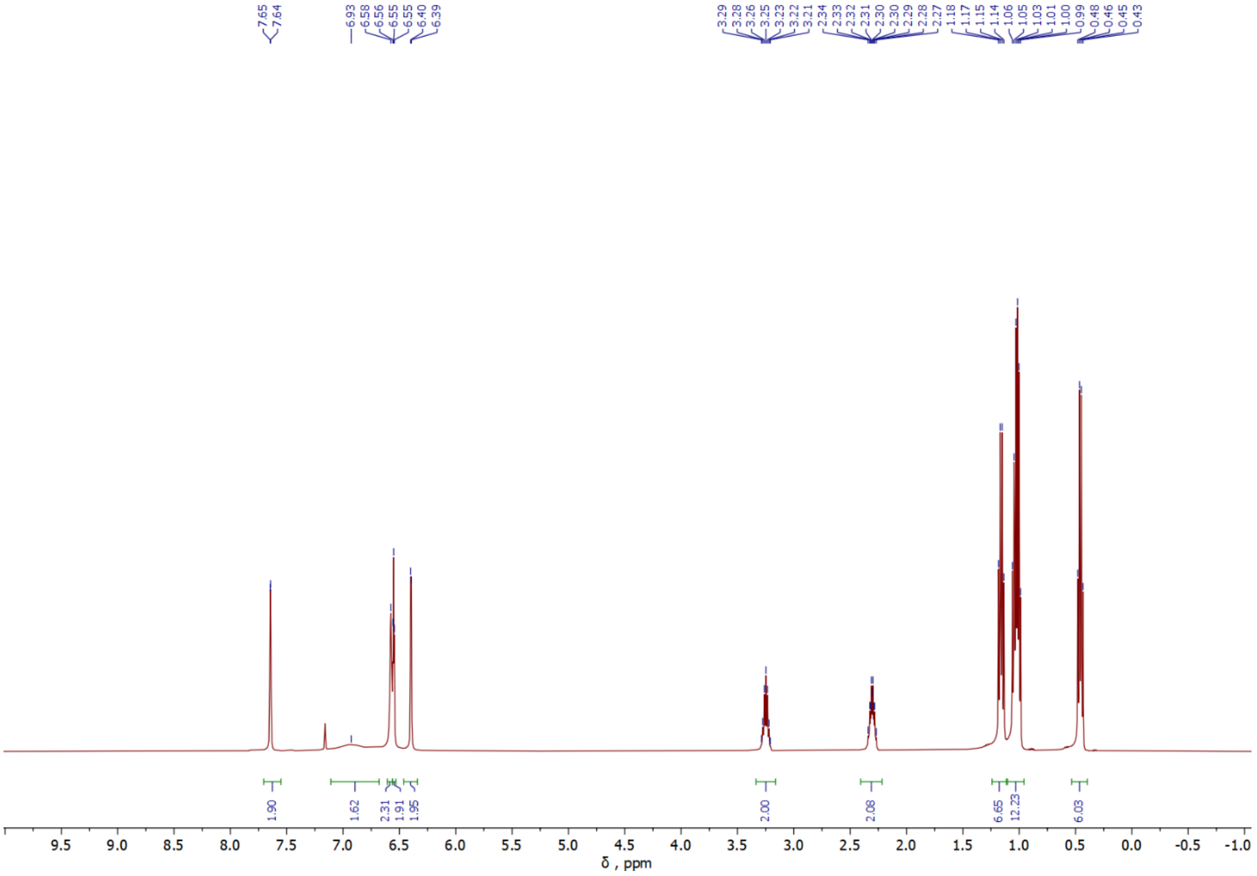

Figure S6. ^{1}H NMR (500 MHz, C_6D_6) spectrum of **3**.

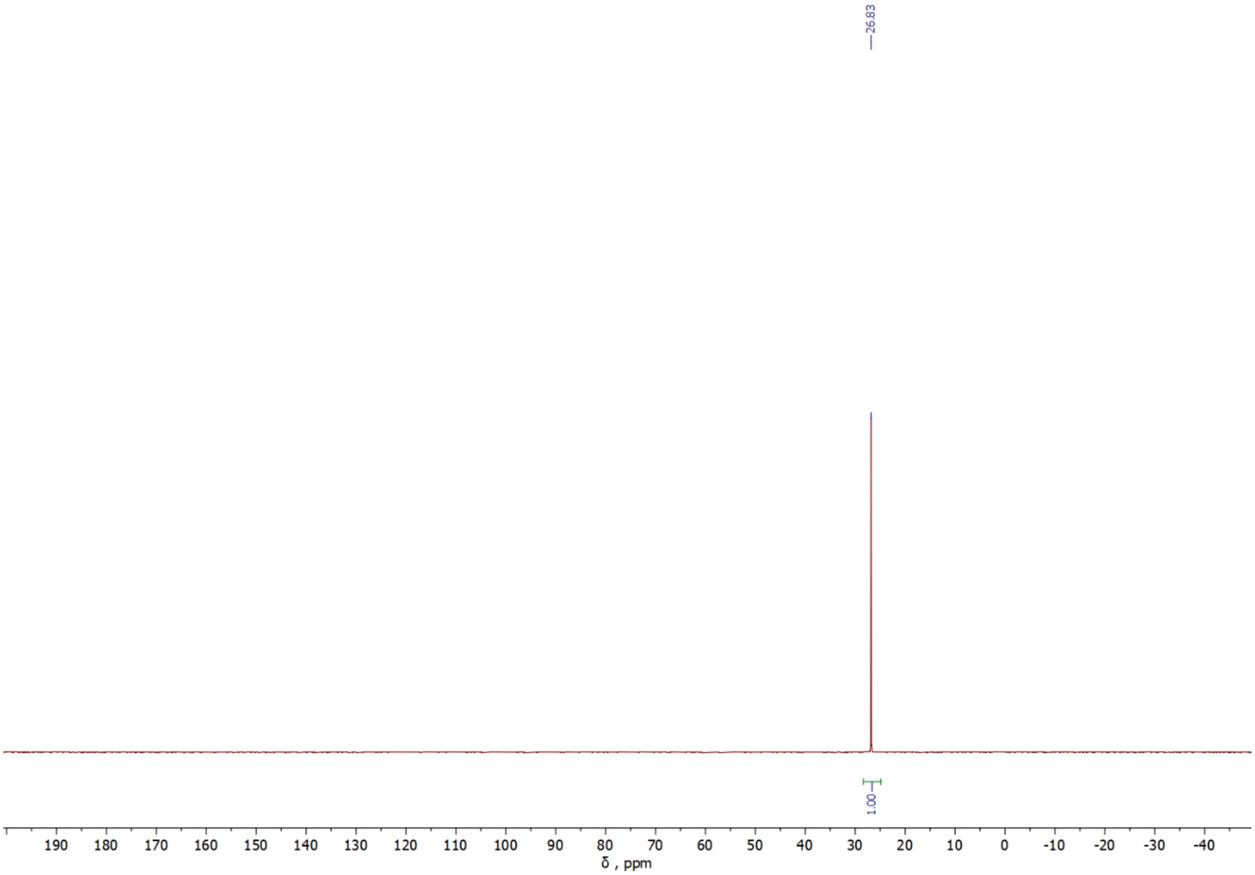

Figure S7. $^{31}P\{^{1}H\}$ NMR (202 MHz, C_6D_6) spectrum of **3**.

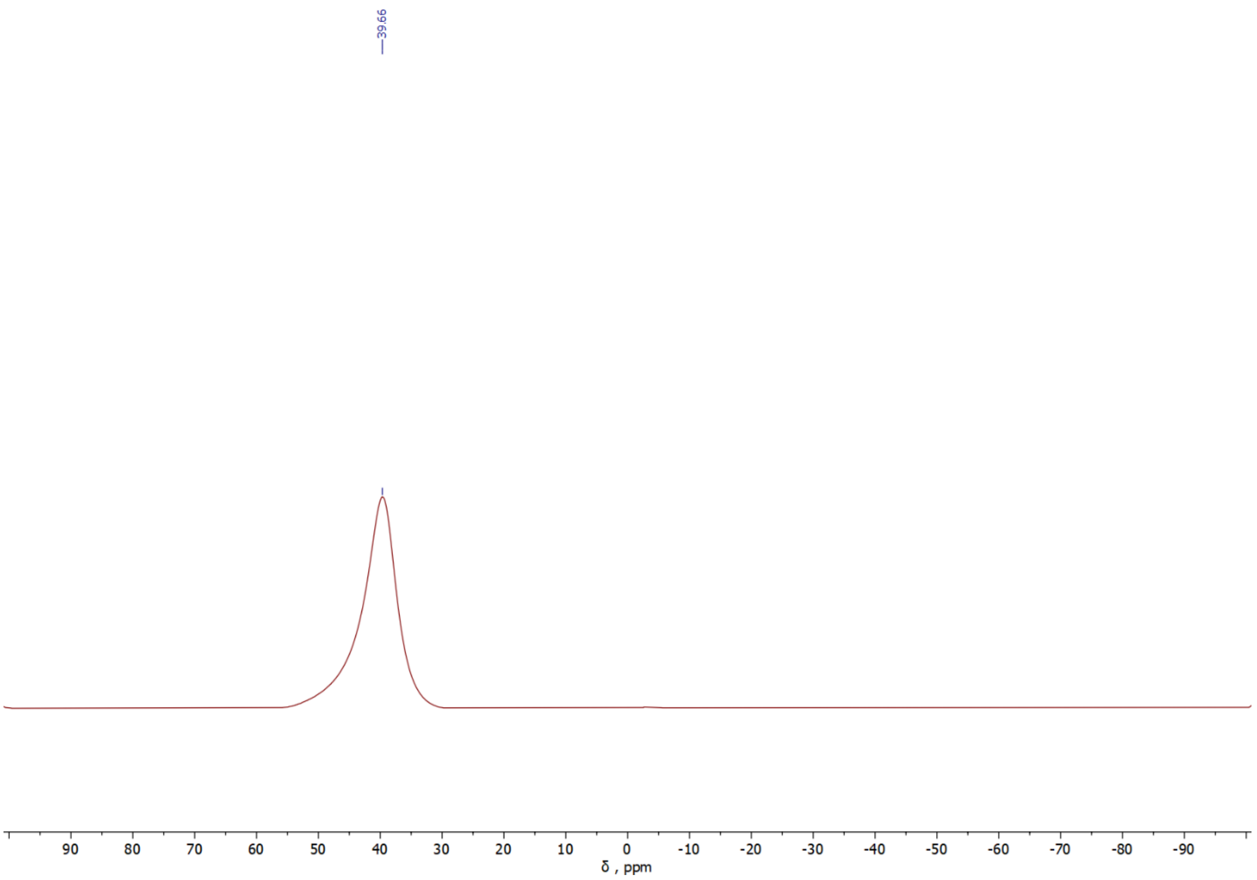

Figure S8. $^{11}B\{^{1}H\}$ NMR (128 MHz, C_6D_6) spectrum of **3**.

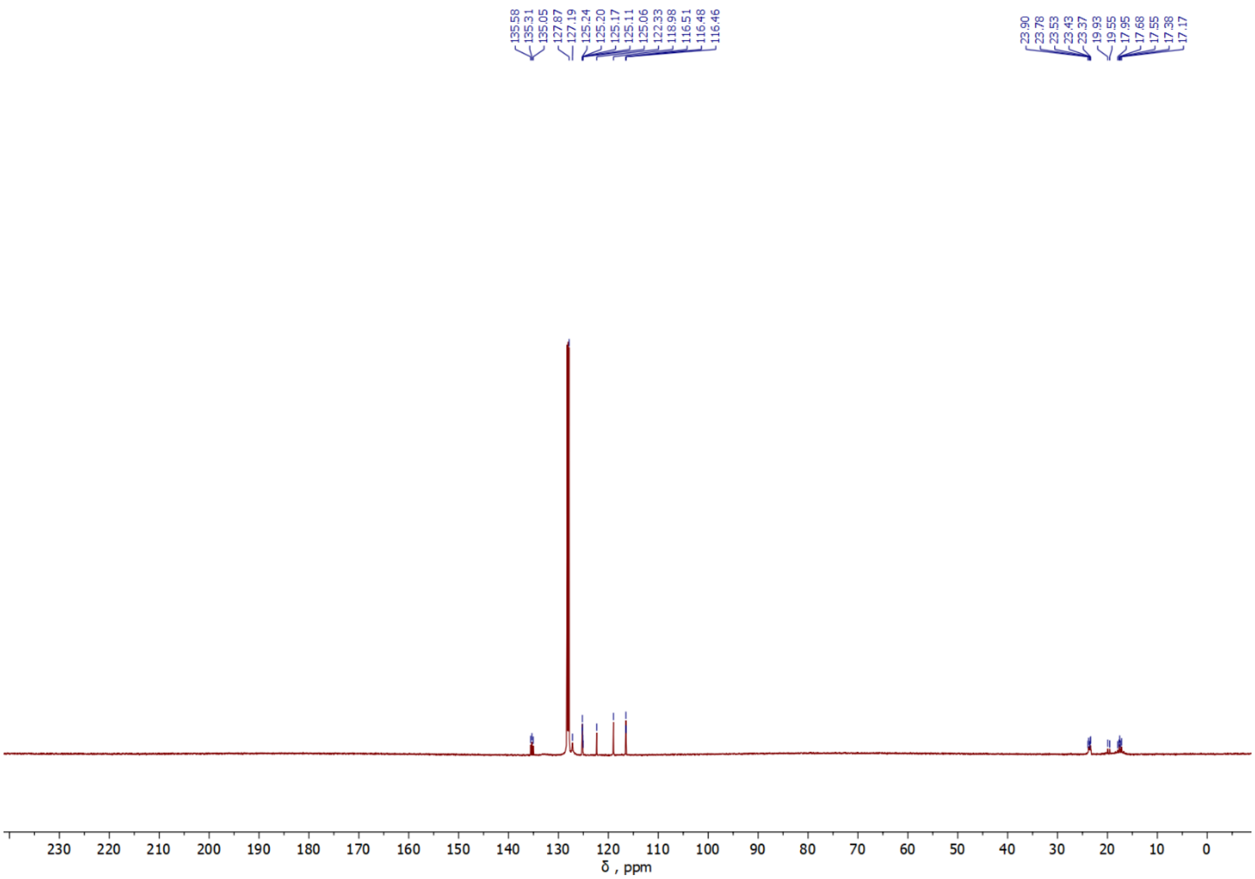

Figure S9. $^{13}C\{^1H\}$ NMR (126 MHz, C_6D_6) spectrum of **3**.

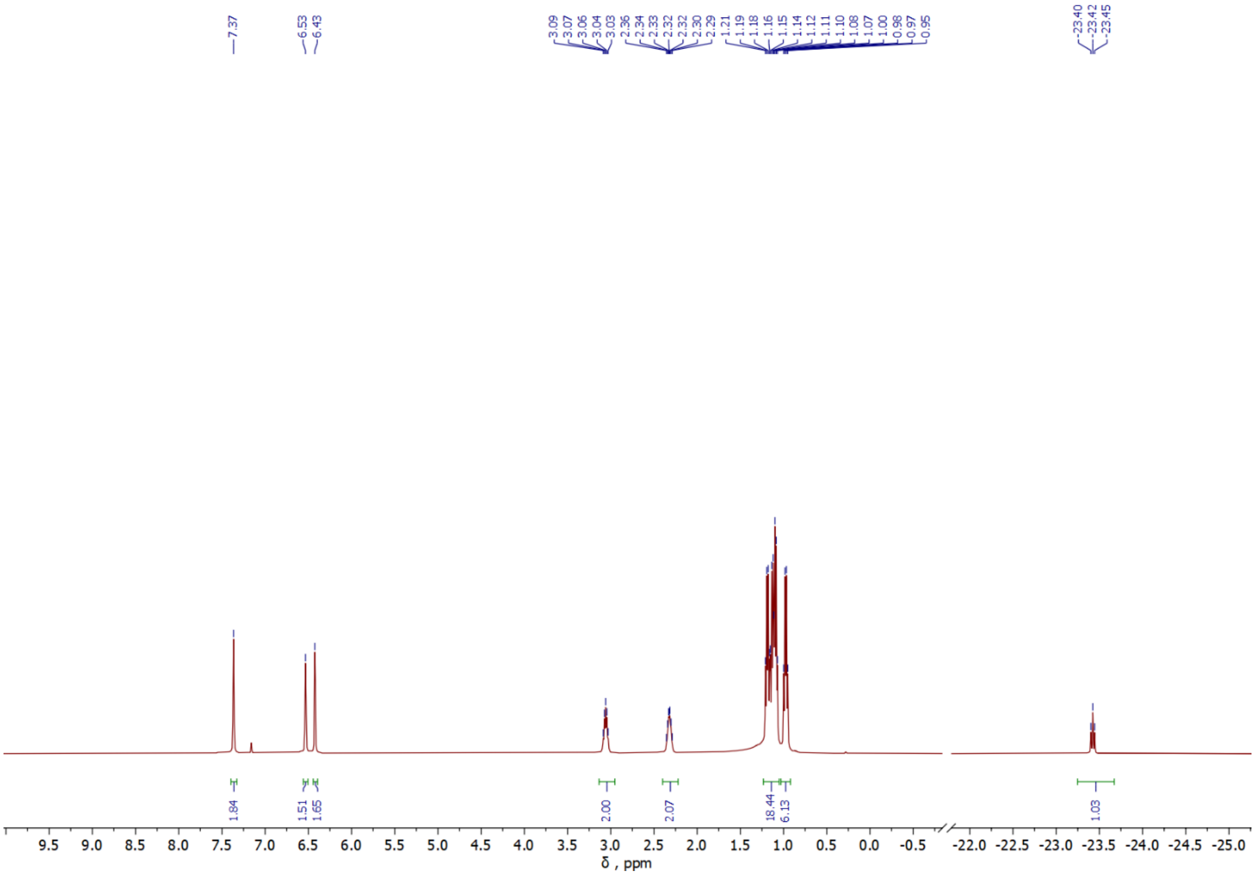

Figure S10. ^{1}H NMR (500 MHz, C_6D_6) spectrum of **4**.

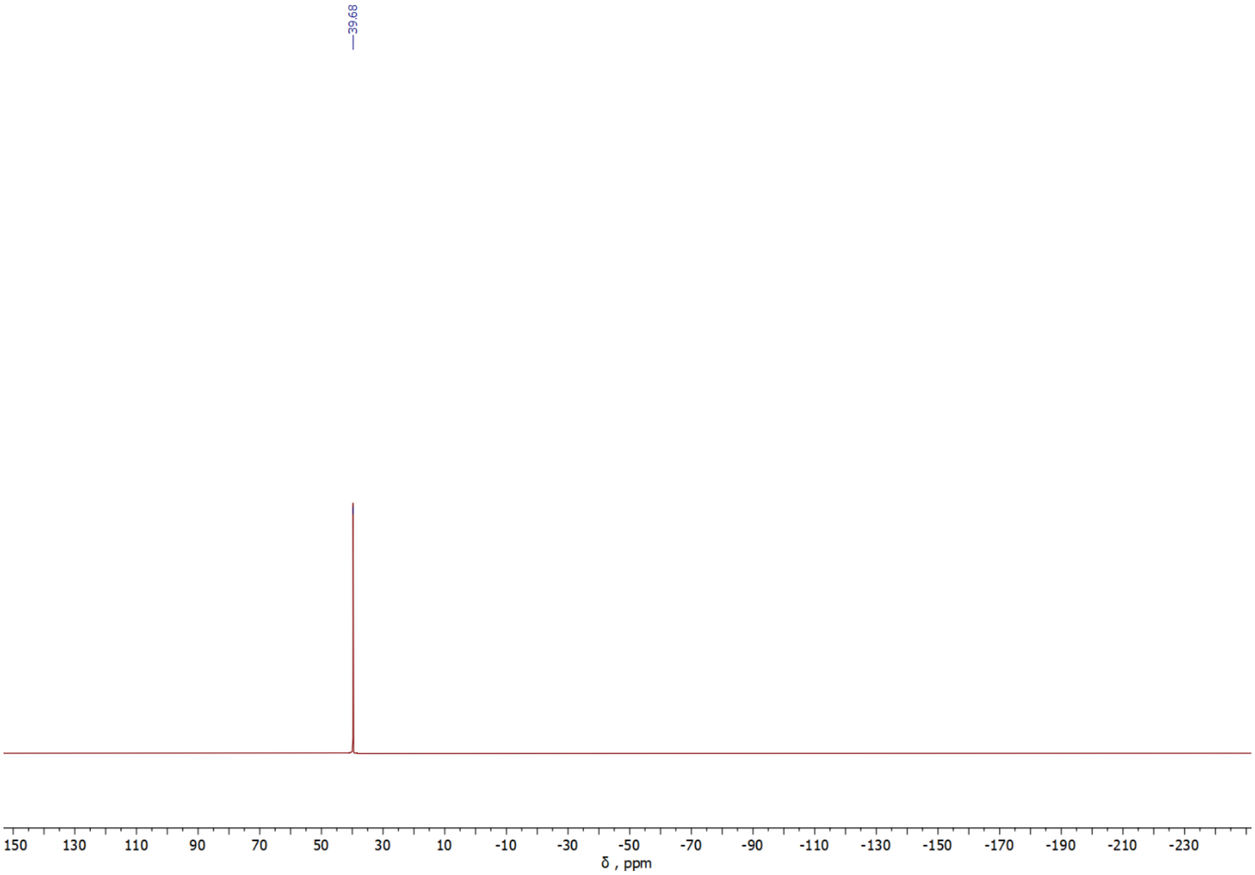

Figure S11. $^{31}P\{^{1}H\}$ NMR (202 MHz, C_6D_6) spectrum of **4**.

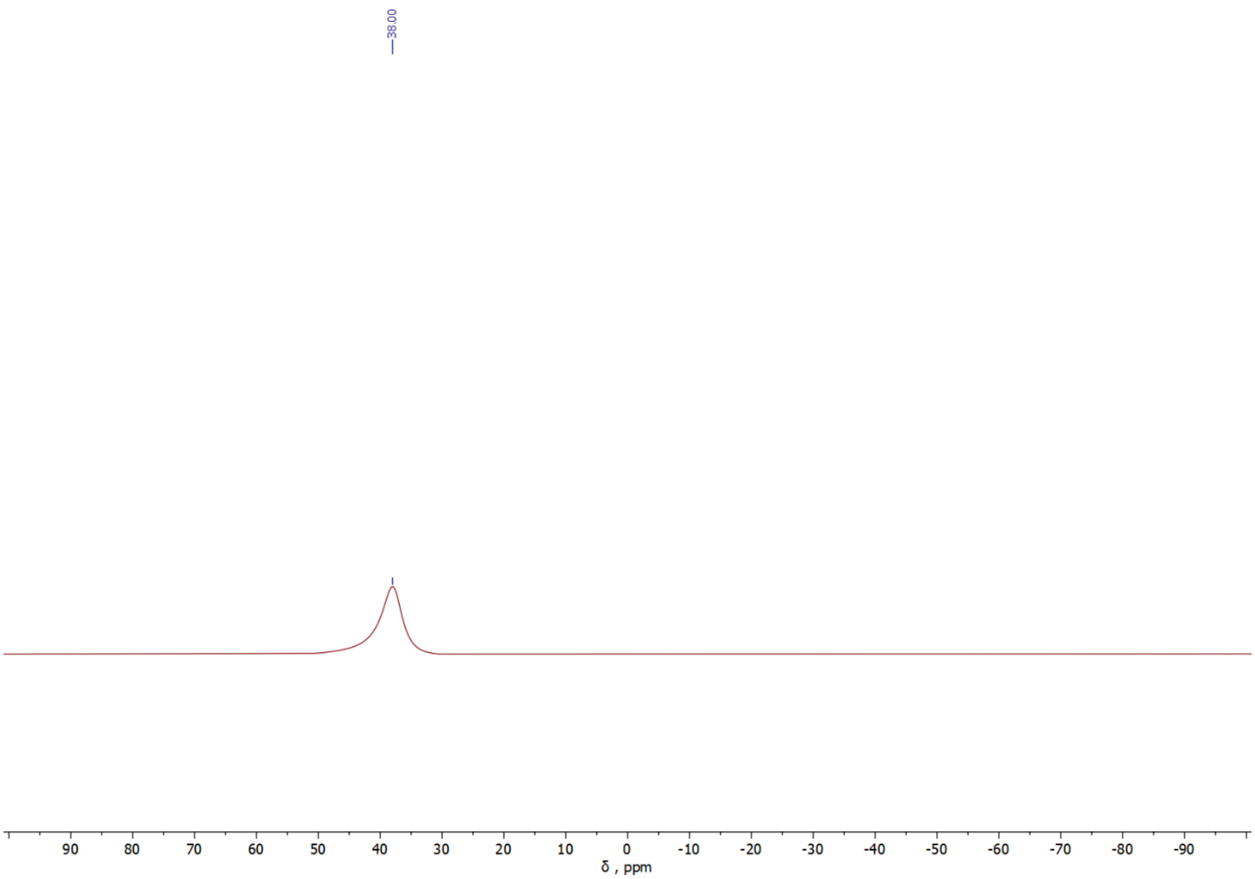

Figure S12. $^{11}B\{^{1}H\}$ NMR (128 MHz, C_6D_6) spectrum of **4**.

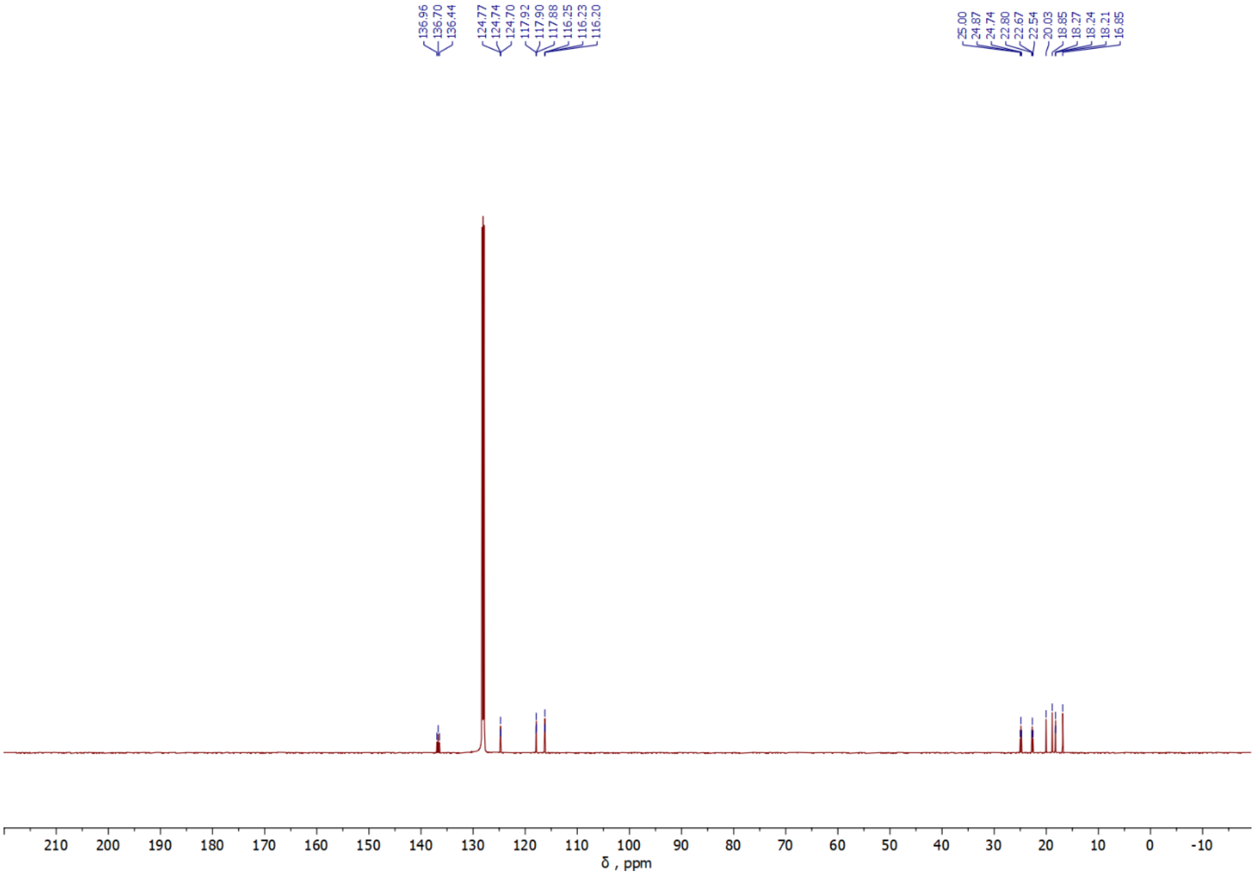

Figure S13. $^{13}C\{^{1}H\}$ NMR (126 MHz, C_6D_6) spectrum of **4**.

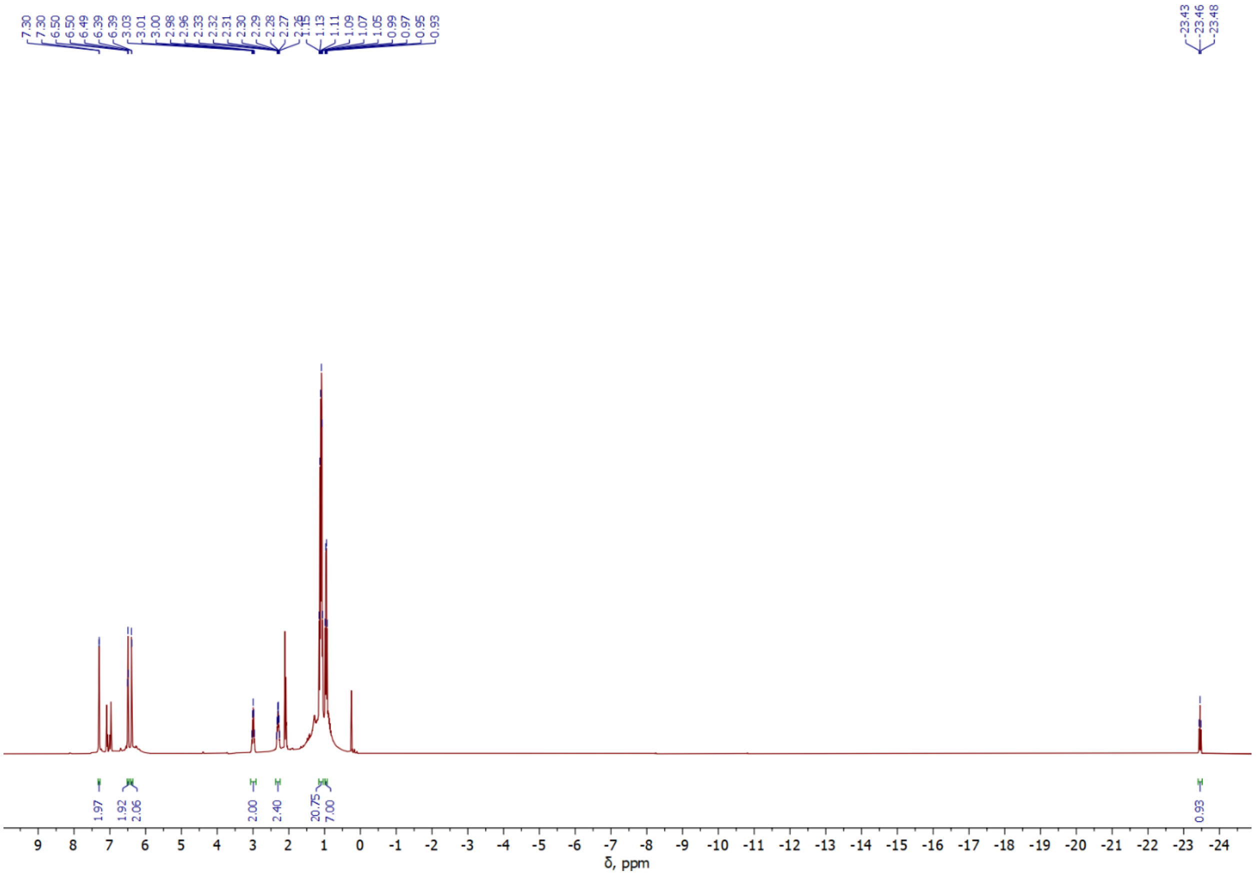

Figure S14. ^{1}H NMR (400 MHz, Toluene-d_8) spectrum of **4**, silicone grease present at δ0.26 ppm.

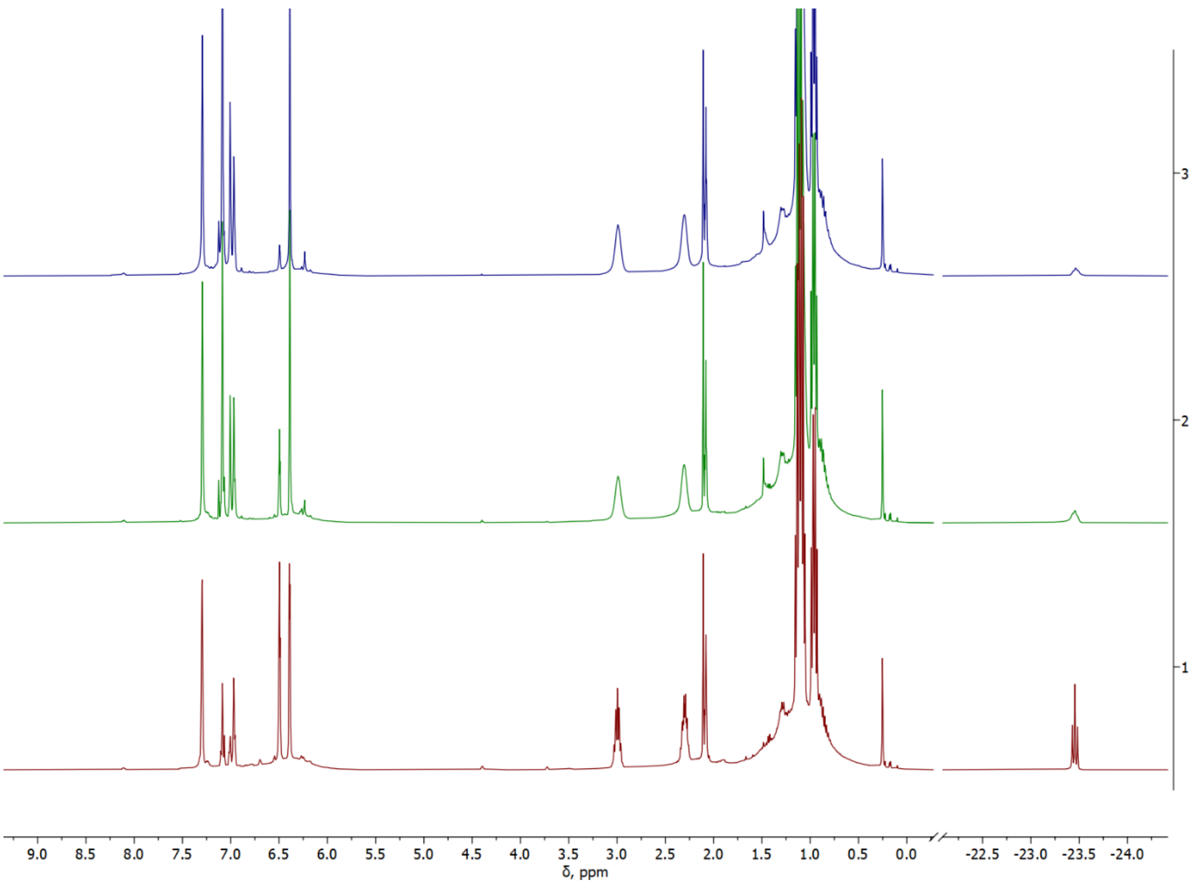

Figure S15. Overlayed ^{1}H NMR (400 MHz, Toluene-d$_8$) spectrum of **4** (bottom), and thermolysis under 1 atm H_2 at 1 hour (middle) and 3 hours (top).

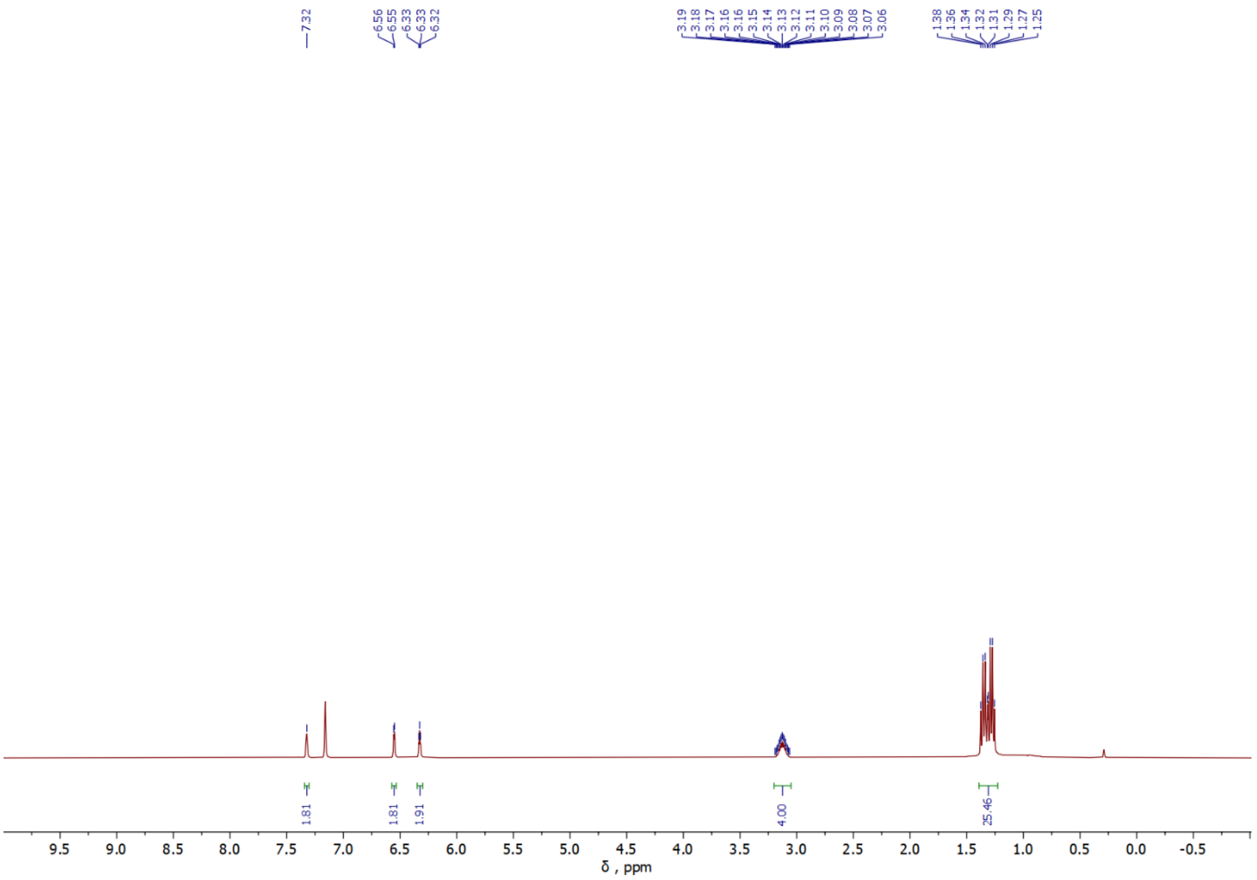

Figure S16. ^{1}H NMR (400 MHz, C_6D_6) spectrum of **5a**.

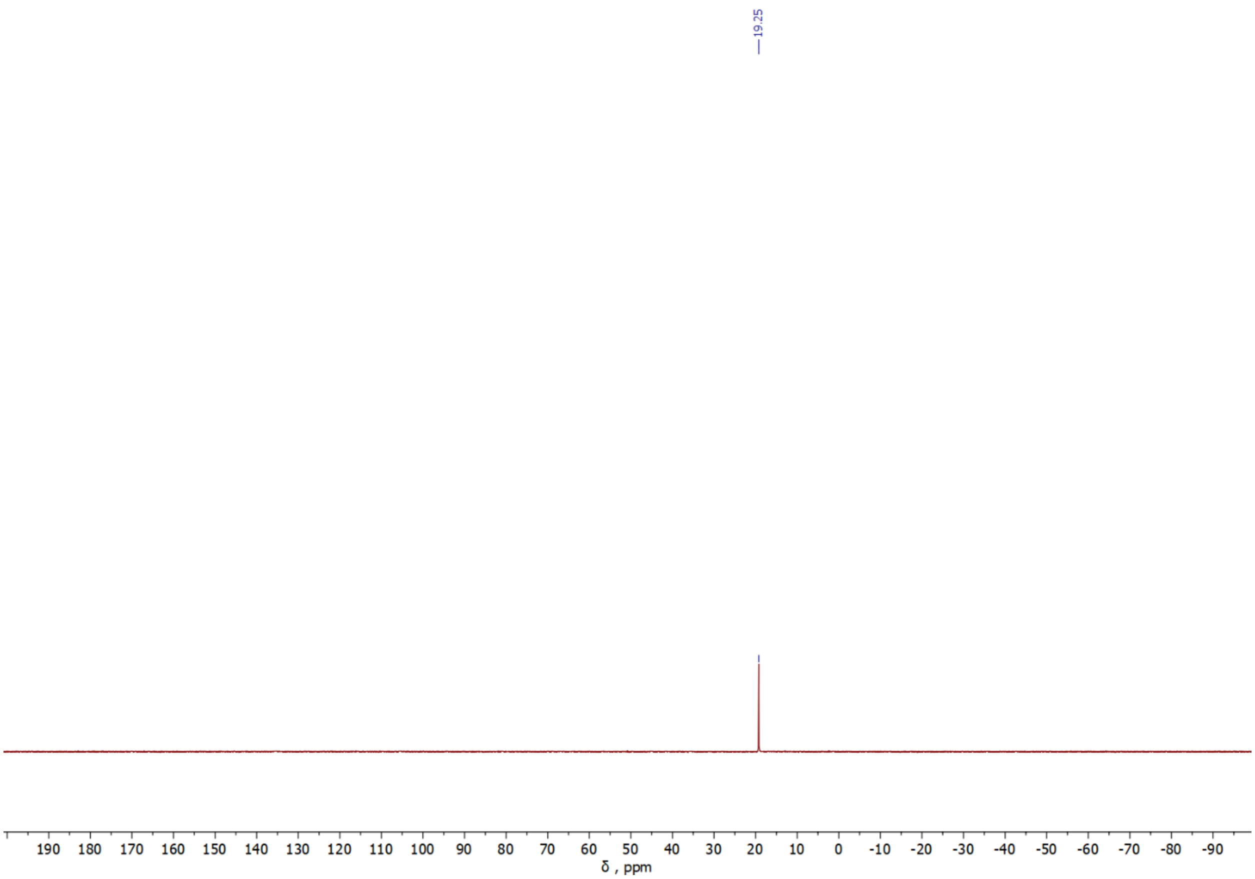

Figure S17. $^{31}P\{^1H\}$ NMR (202 MHz, C_6D_6) spectrum of **5a**.

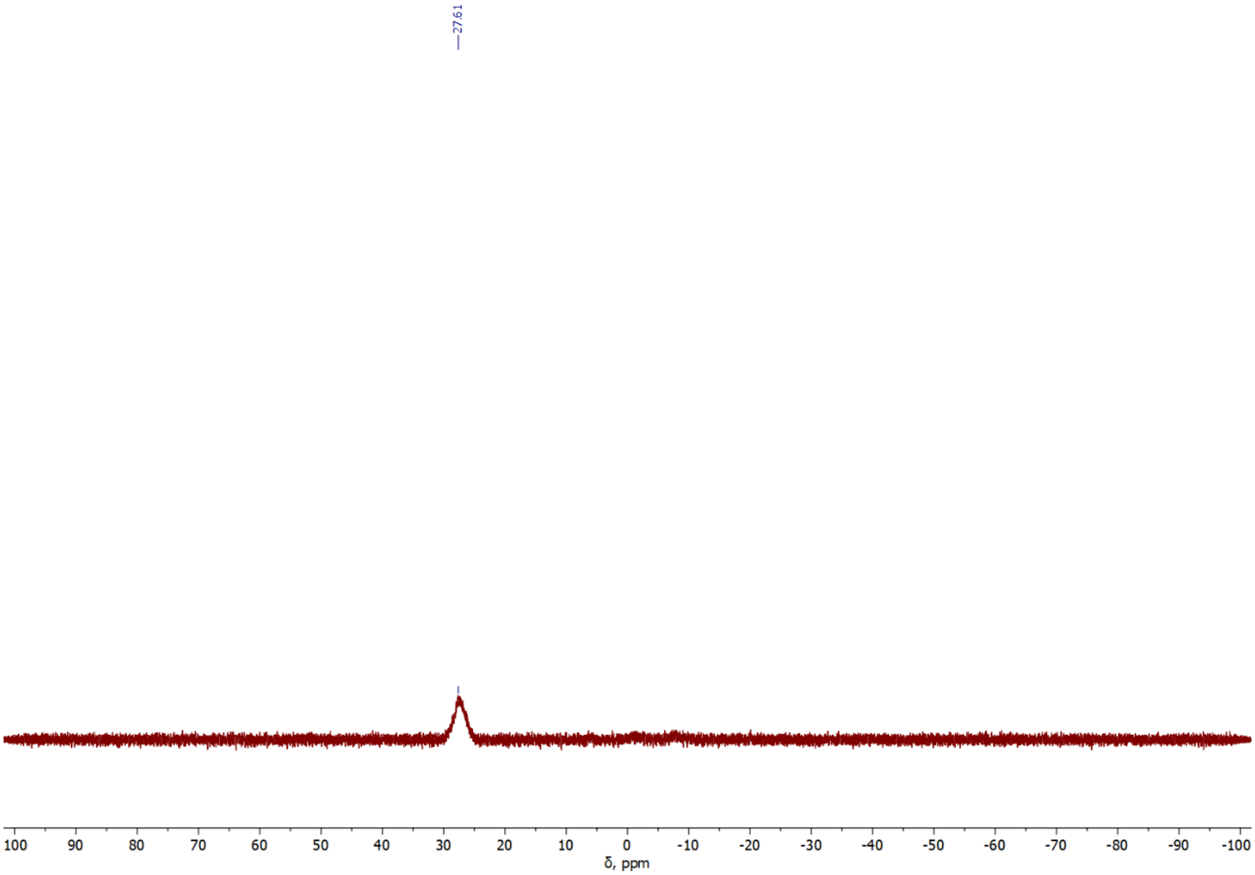

Figure S18. $^{11}B\{^1H\}$ NMR (160 MHz, C_6D_6) spectrum of **5a**.

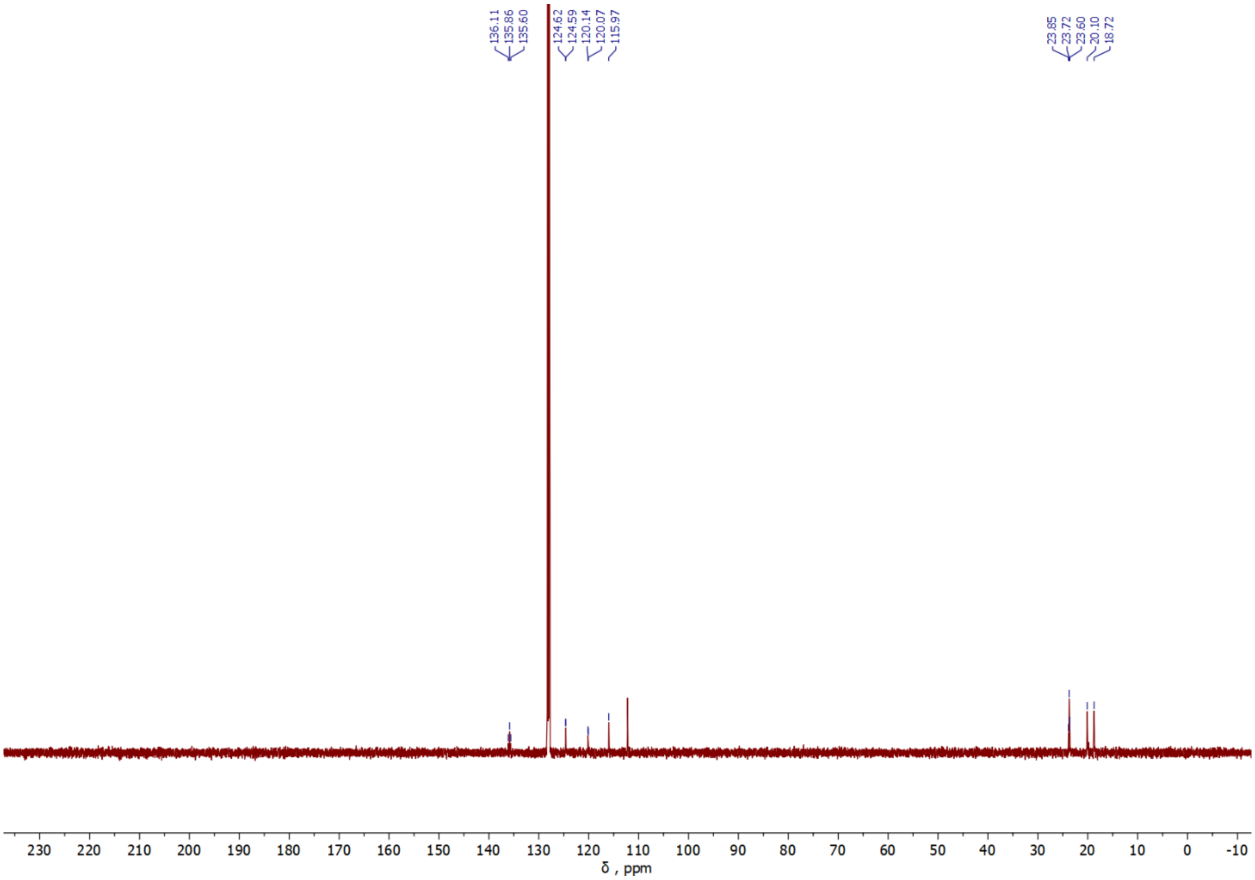

Figure S19. $^{13}C\{^{1}H\}$ NMR (126 MHz, C_6D_6) spectrum of **5a**.

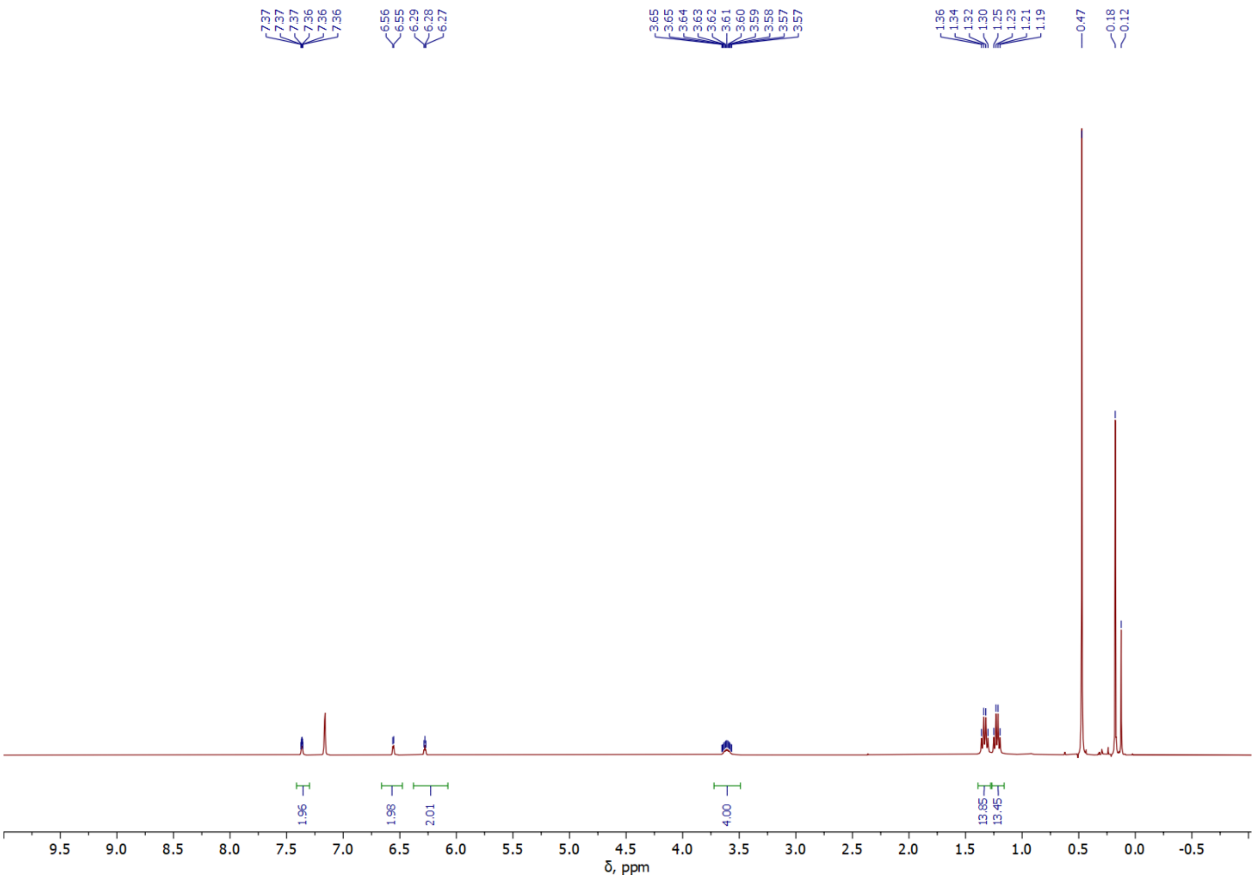

Figure S20. ^{1}H NMR (400 MHz, C_6D_6) spectrum of in situ generated **5b**. Excess TMSI present at δ 0.47, TMSCl byproduct at δ 0.18, and HMDSO at δ 0.12.

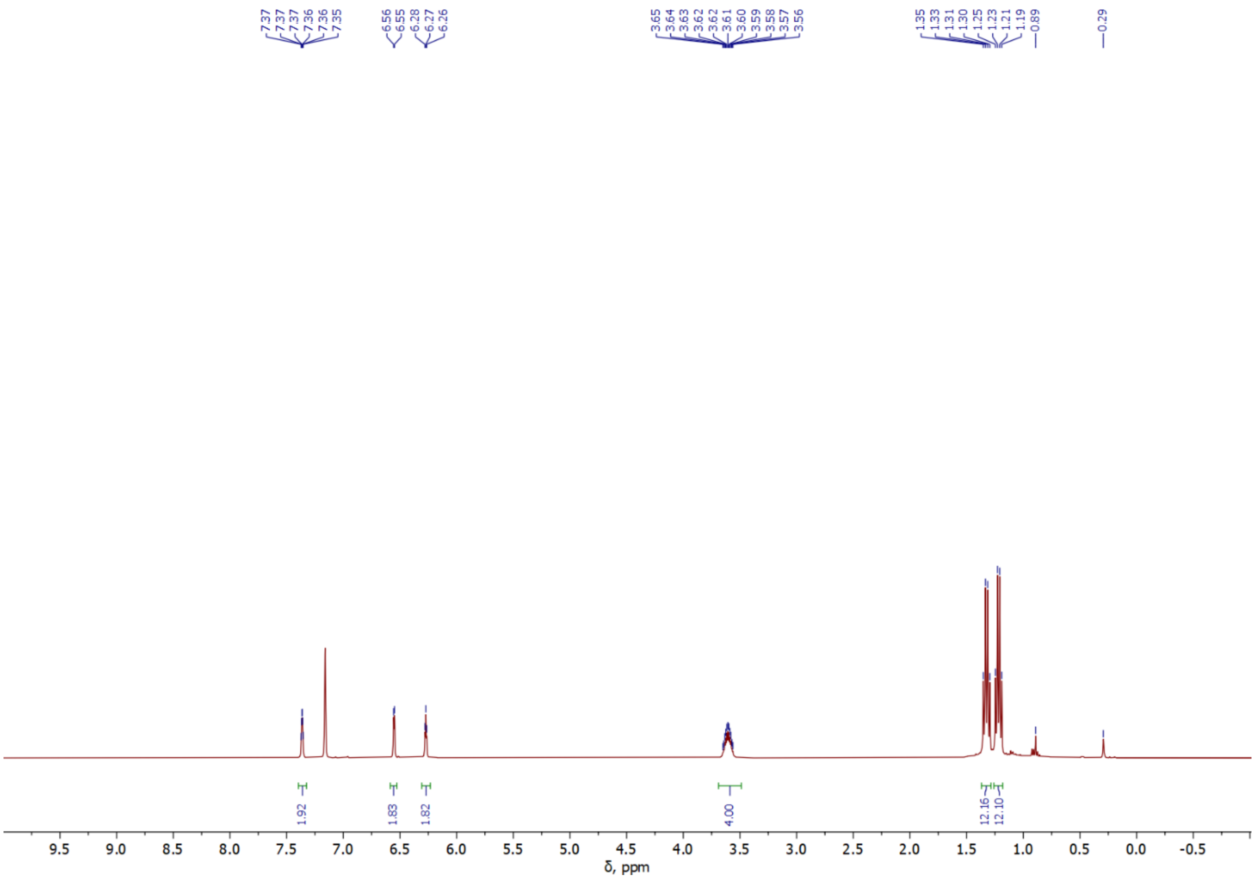

Figure S21. ^{1}H NMR (400 MHz, C_6D_6) spectrum of **5b**, pentane and silicone grease present at δ 0.89, 0.29, respectively.

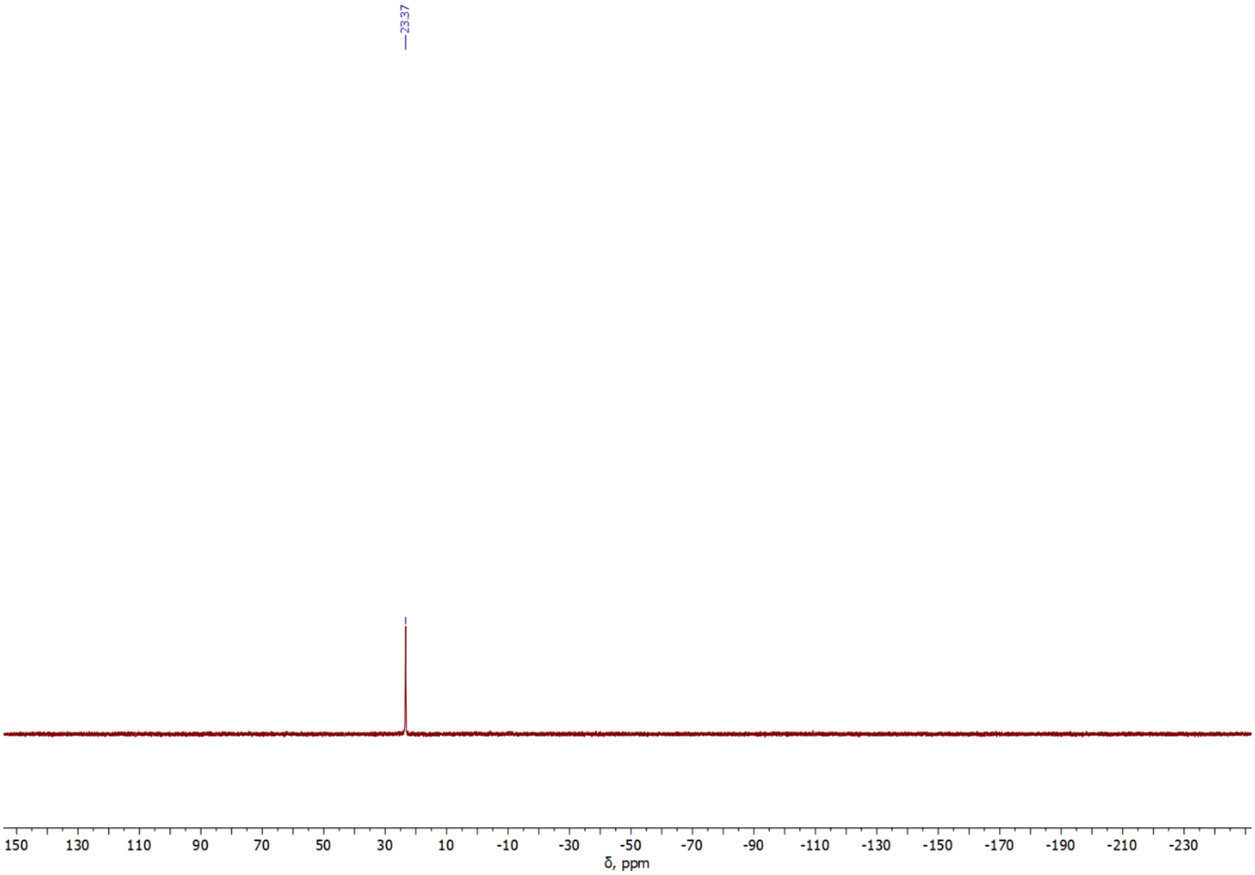

Figure S22. $^{31}P\{^{1}H\}$ NMR (162 MHz, C_6D_6) spectrum of **5b**.

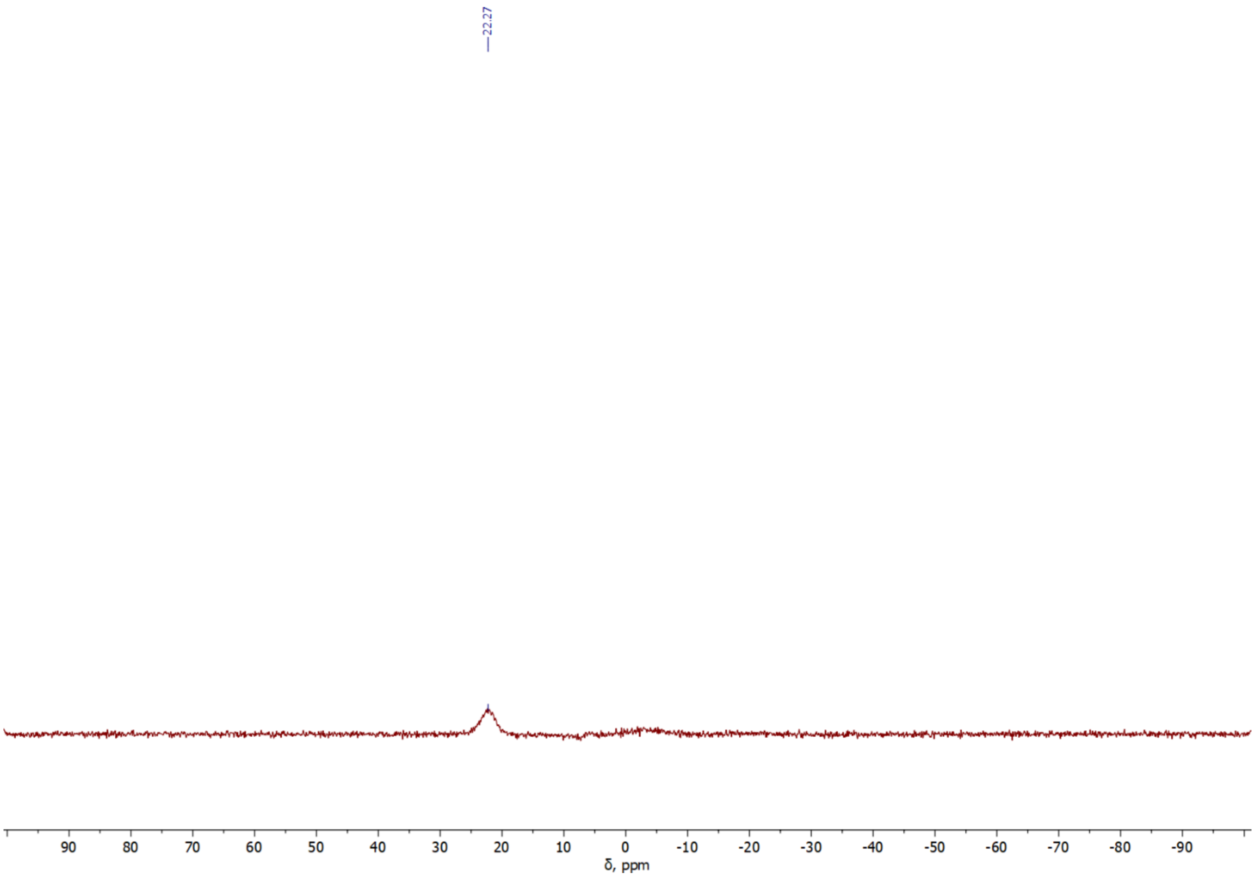

Figure S23. $^{11}B\{^{1}H\}$ NMR (128 MHz, C_6D_6) spectrum of **5b**.

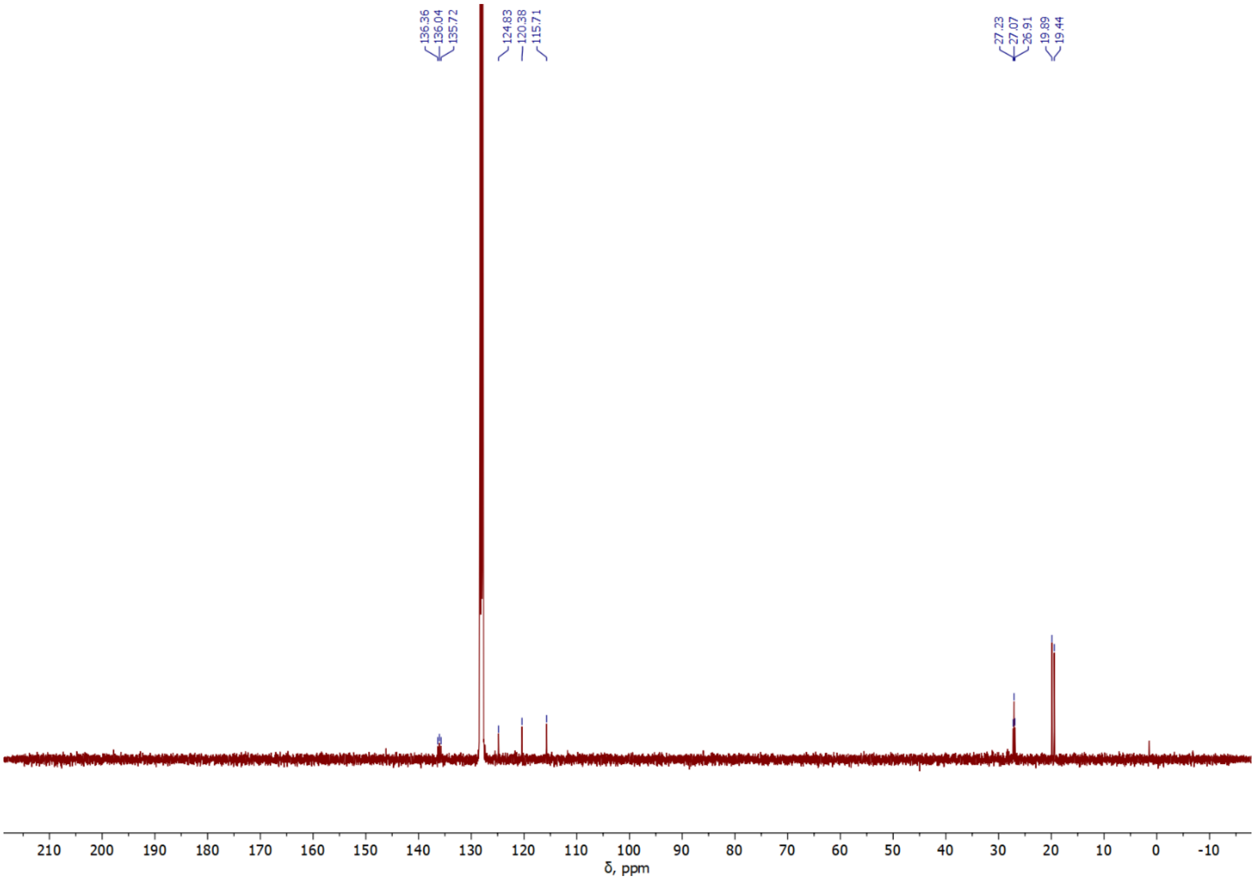

Figure S24. $^{13}C\{^{1}H\}$ NMR (101 MHz, C_6D_6) spectrum of **5b**.

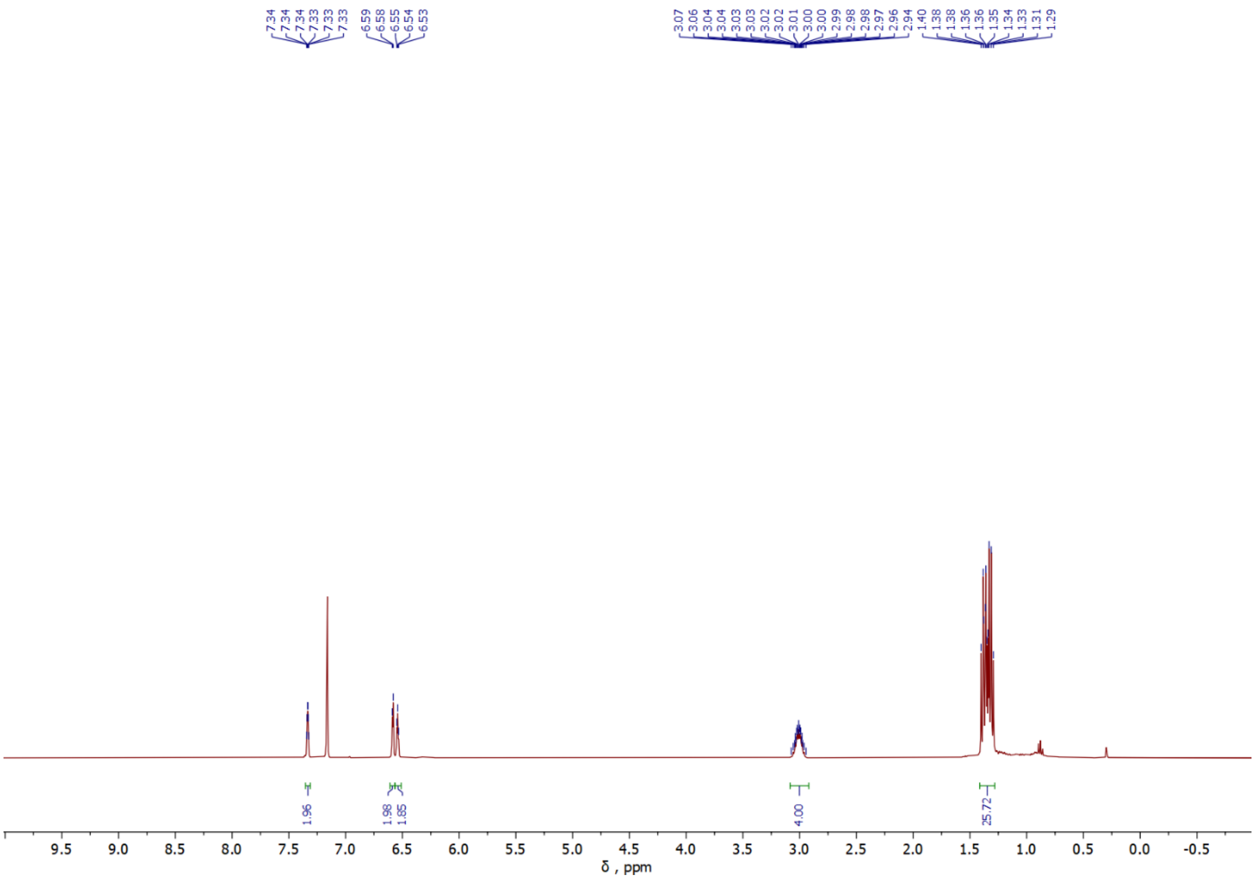

Figure S25. ¹H NMR (400 MHz, C_6D_6) spectrum of **6a**.

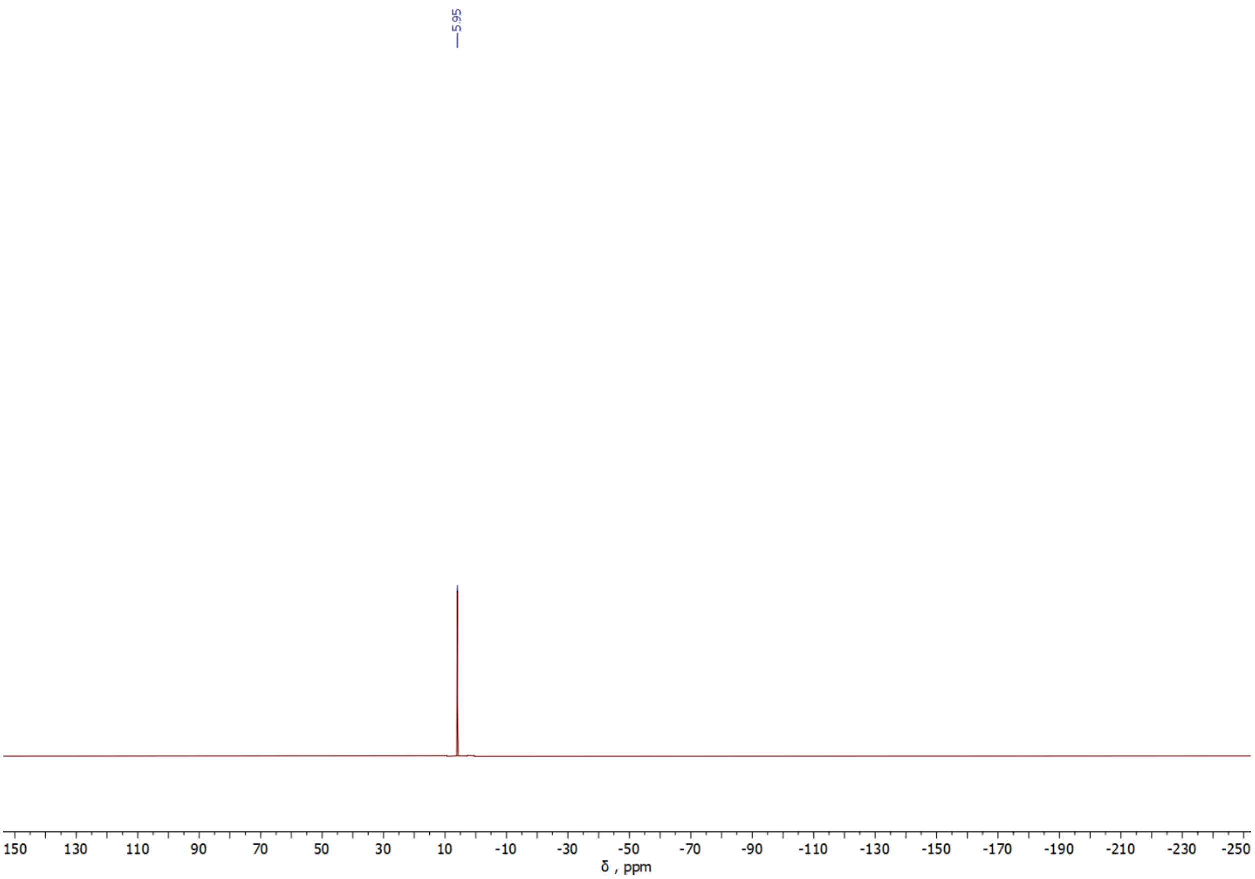

Figure S26. $^{31}P\{^{1}H\}$ NMR (162 MHz, C_6D_6) spectrum of **6a**.

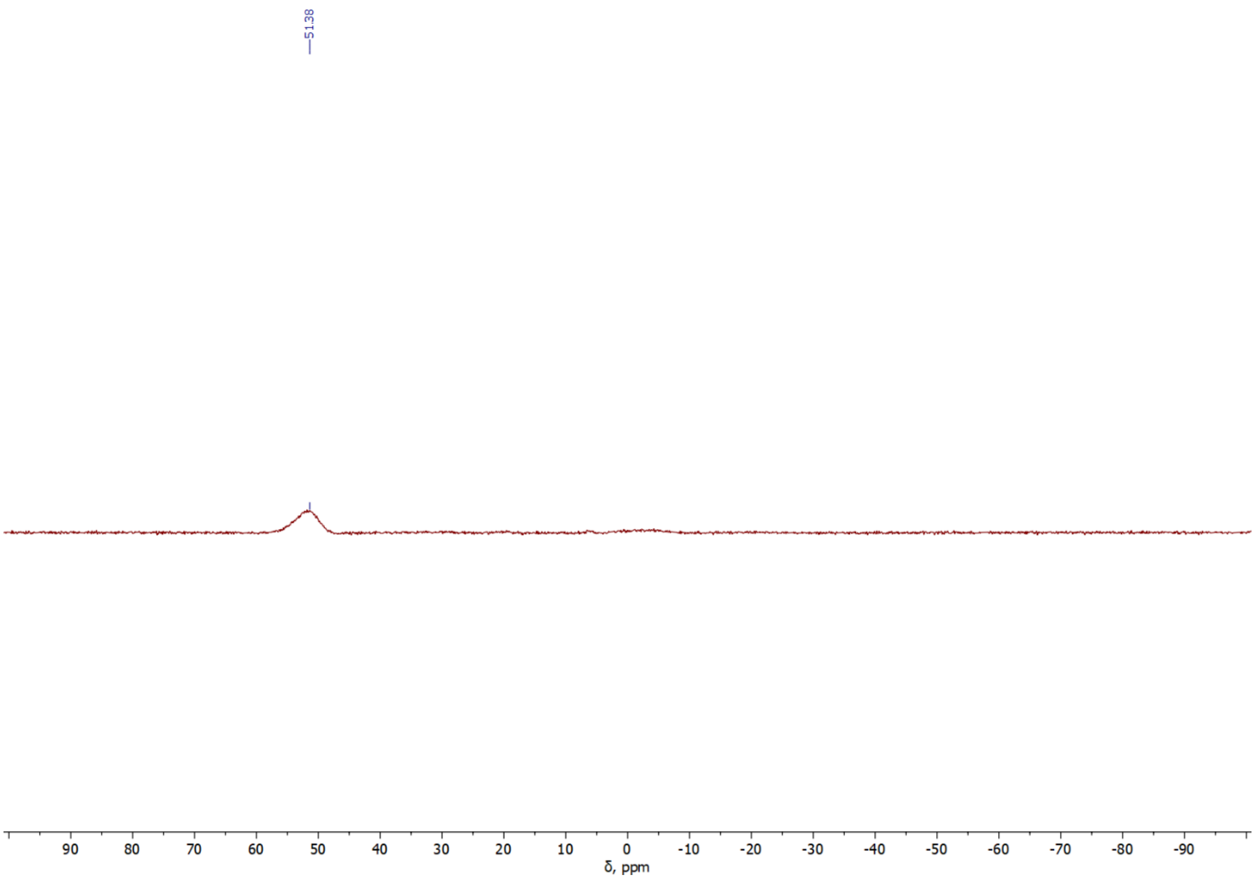

Figure S27. $^{11}B\{^{1}H\}$ NMR (128 MHz, C_6D_6) spectrum of **6a**.

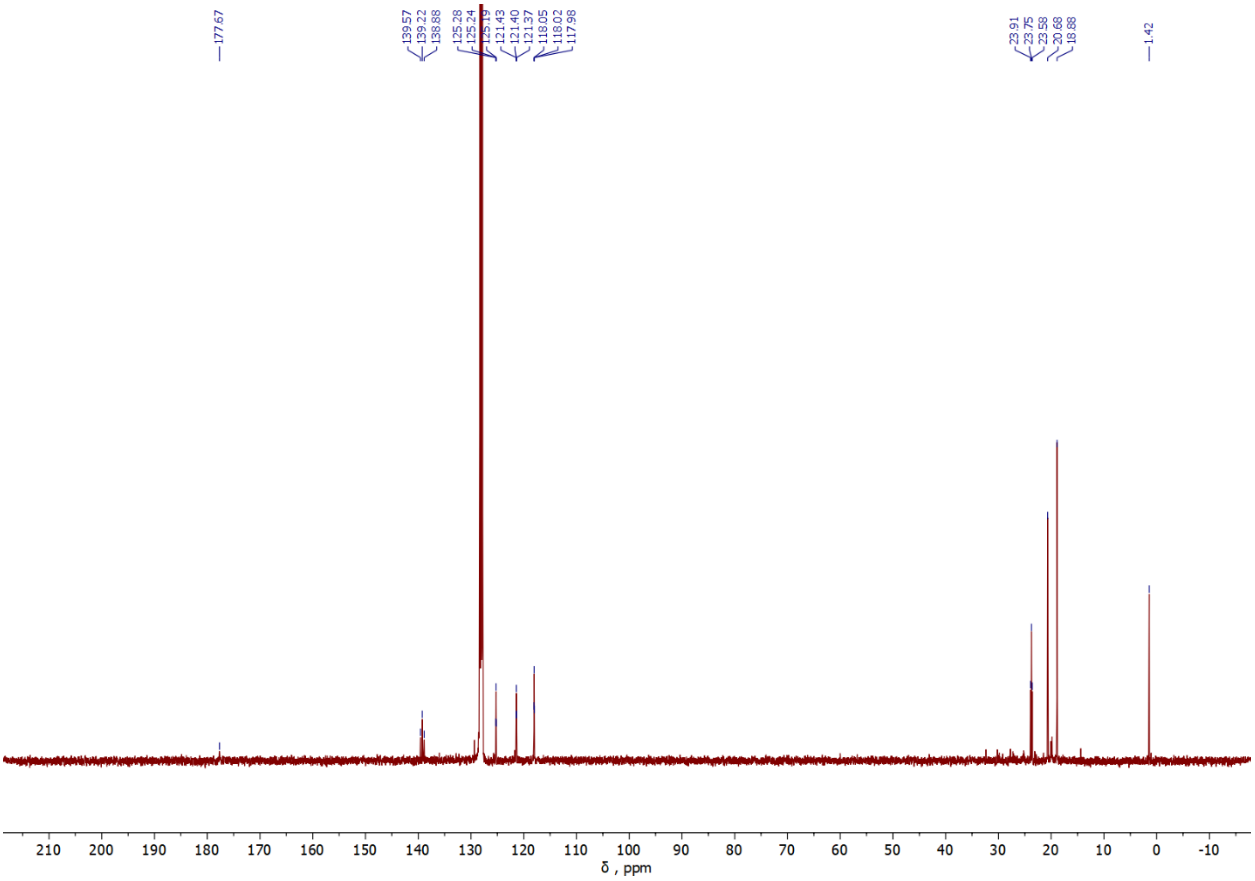

Figure S28. $^{13}C\{^{1}H\}$ NMR (101 MHz, C_6D_6) spectrum of **6a**.

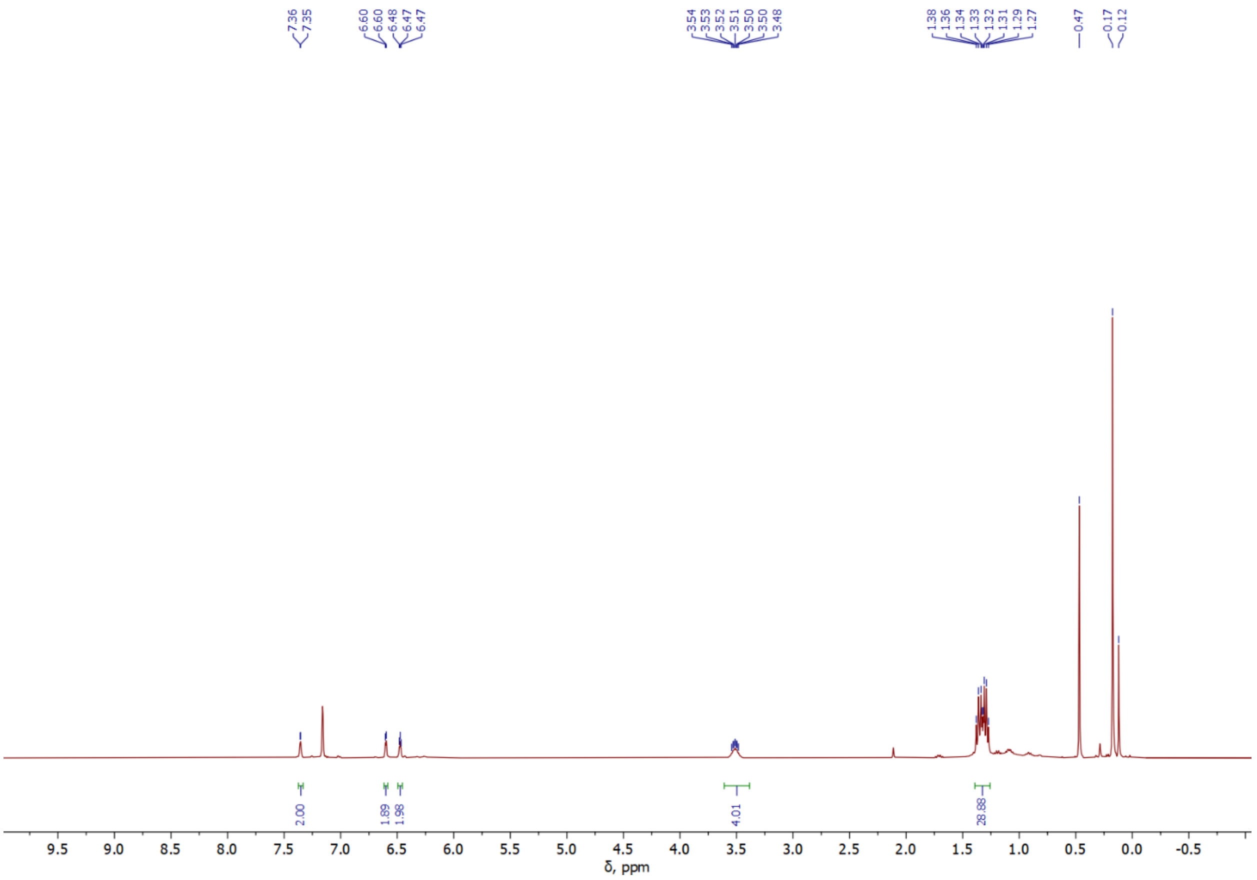

Figure S29. ^{1}H NMR (400 MHz, C_6D_6) spectrum of **6b**.

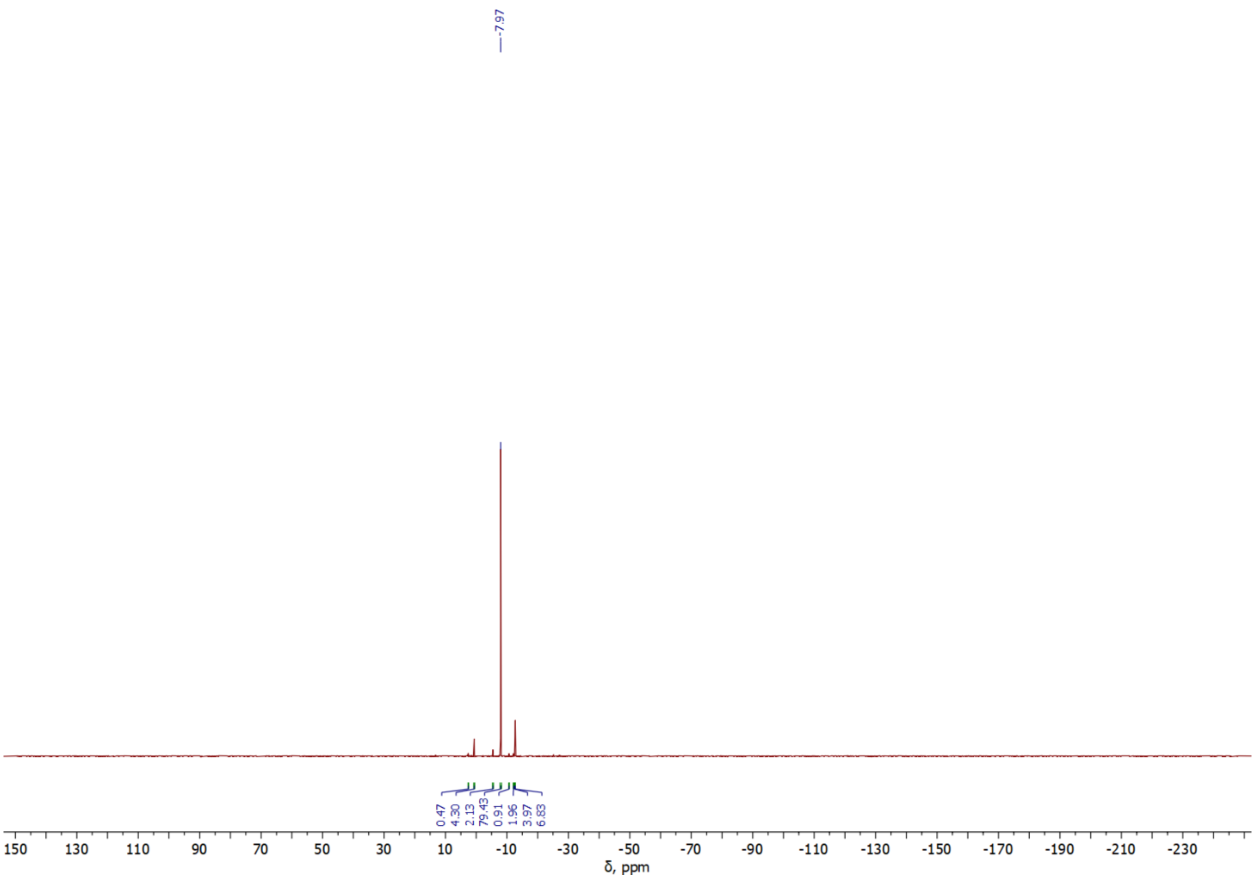

Figure S30. $^{31}P\{^{1}H\}$ NMR (162 MHz, C_6D_6) spectrum of **6b**.

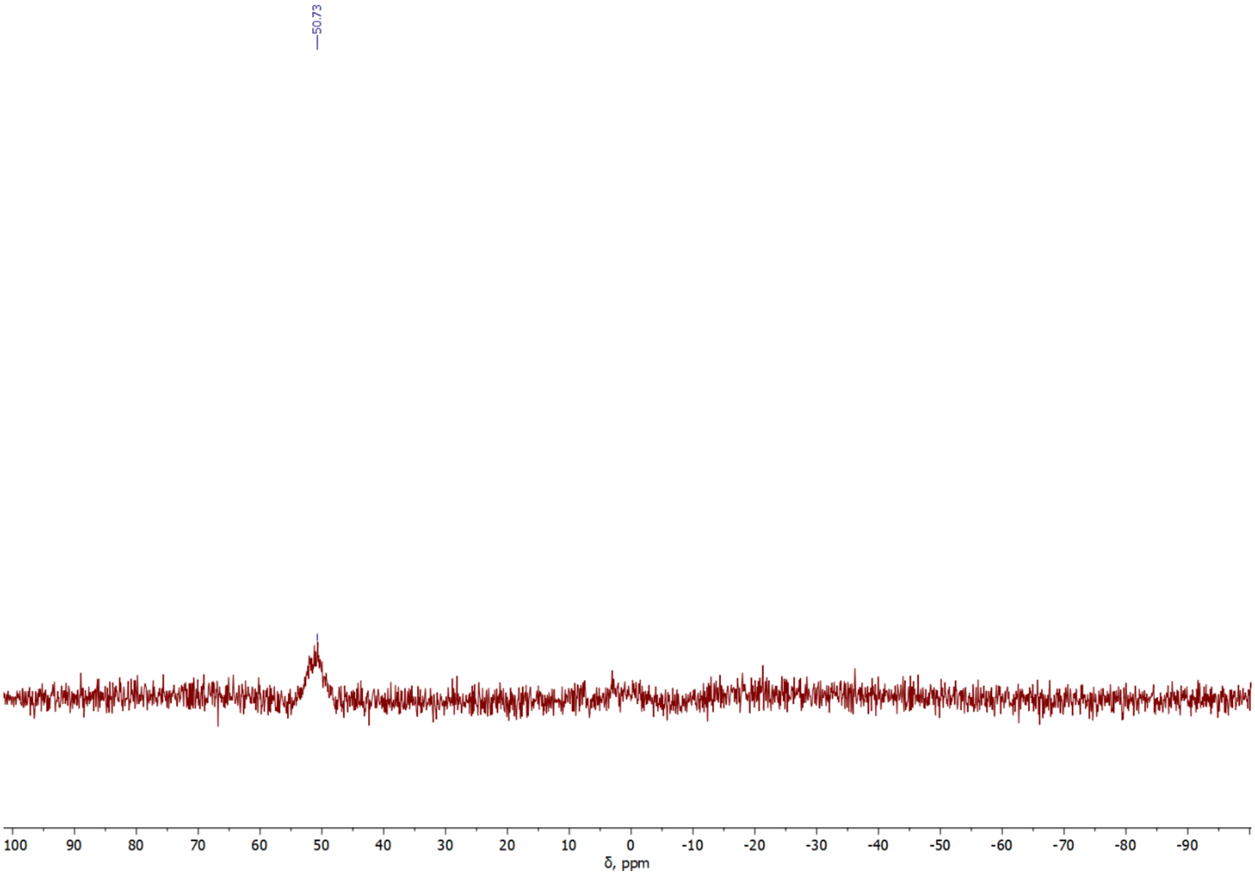

Figure S31. $^{11}B\{^{1}H\}$ NMR (128 MHz, C_6D_6) spectrum of **6b**.

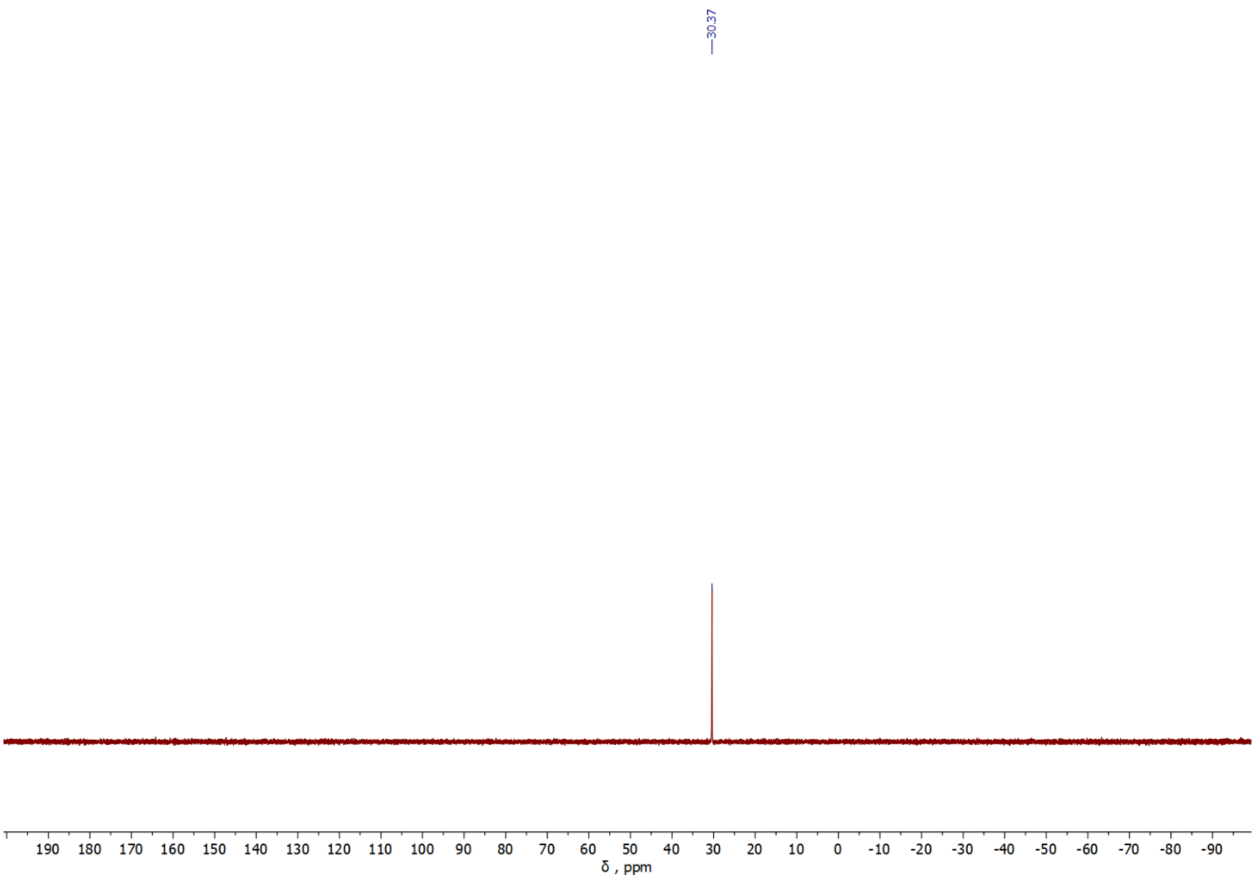

Figure S32. $^{31}P\{^{1}H\}$ NMR (202 MHz, THF) spectrum of **7**, degassed before workup.

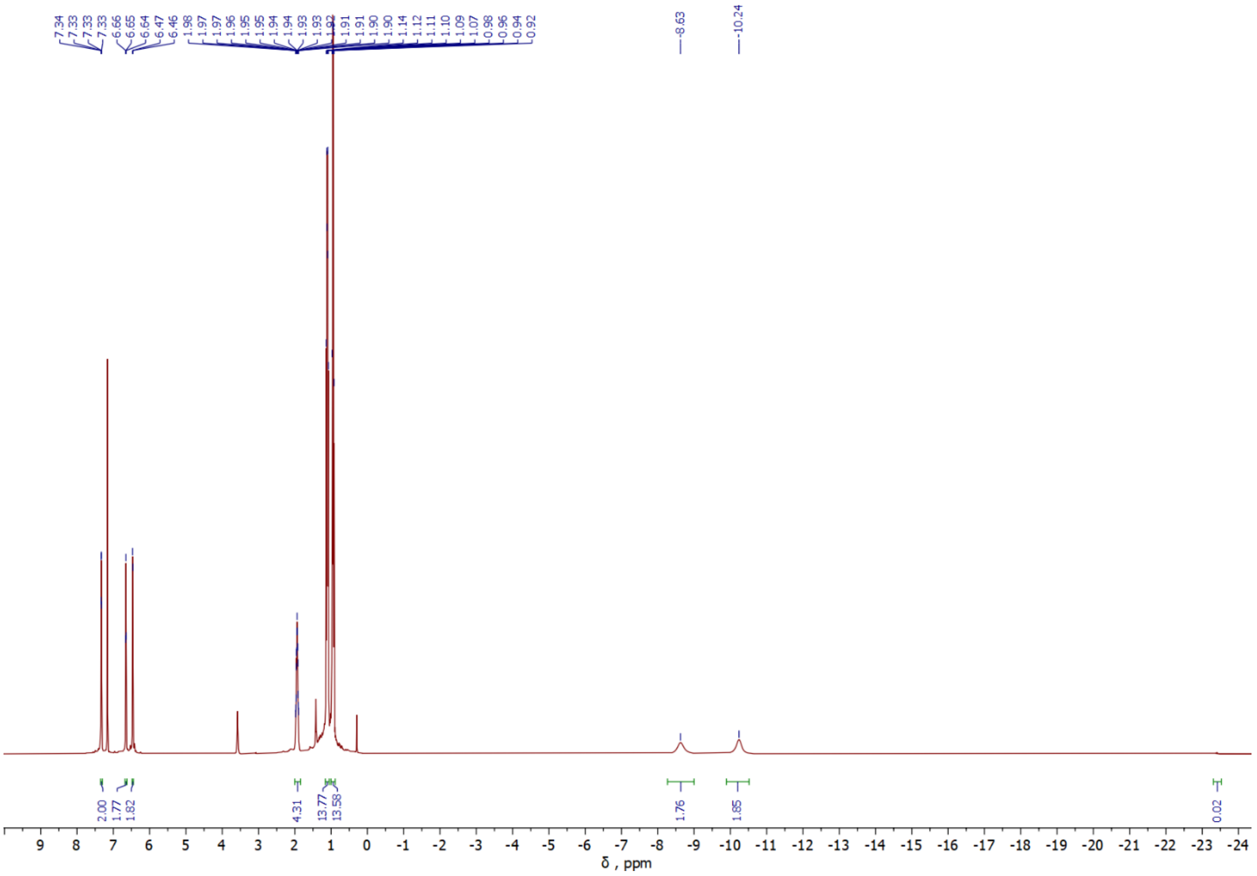

Figure S33. ^{1}H NMR (500 MHz, C_6D_6) spectrum of **7** (98%, 2% of **4** denoted by [*]).

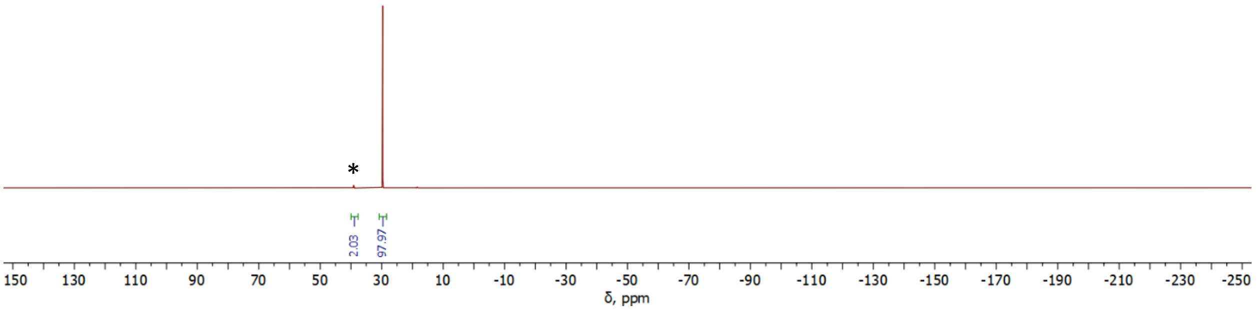

Figure S34. $^{31}P\{^{1}H\}$ NMR (162 MHz, C_6D_6) spectrum of **7** (98%, 2% of **4** denoted by [*]).

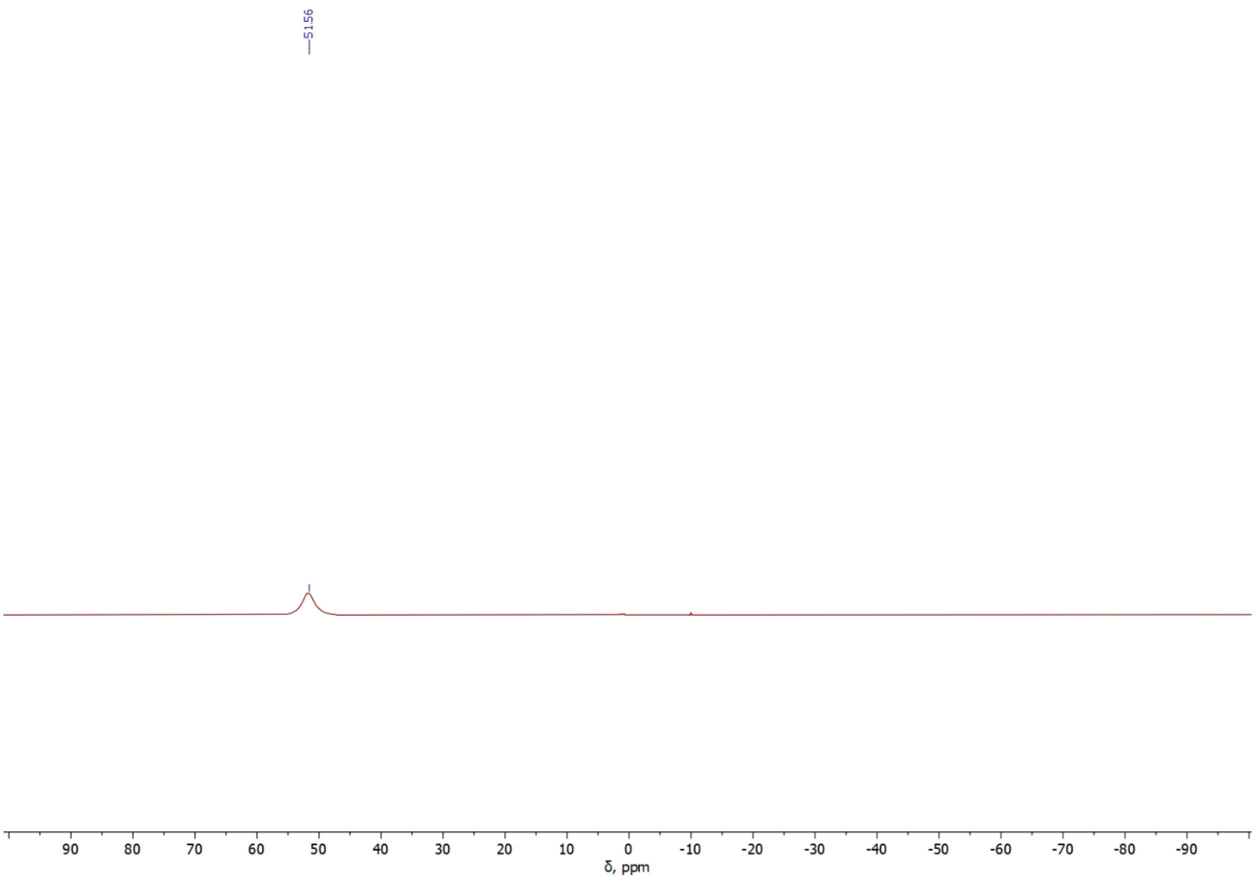

Figure S35. $^{11}B\{^{1}H\}$ NMR (128 MHz, C_6D_6) spectrum of **7** (98%, 2% of **4** not detected).

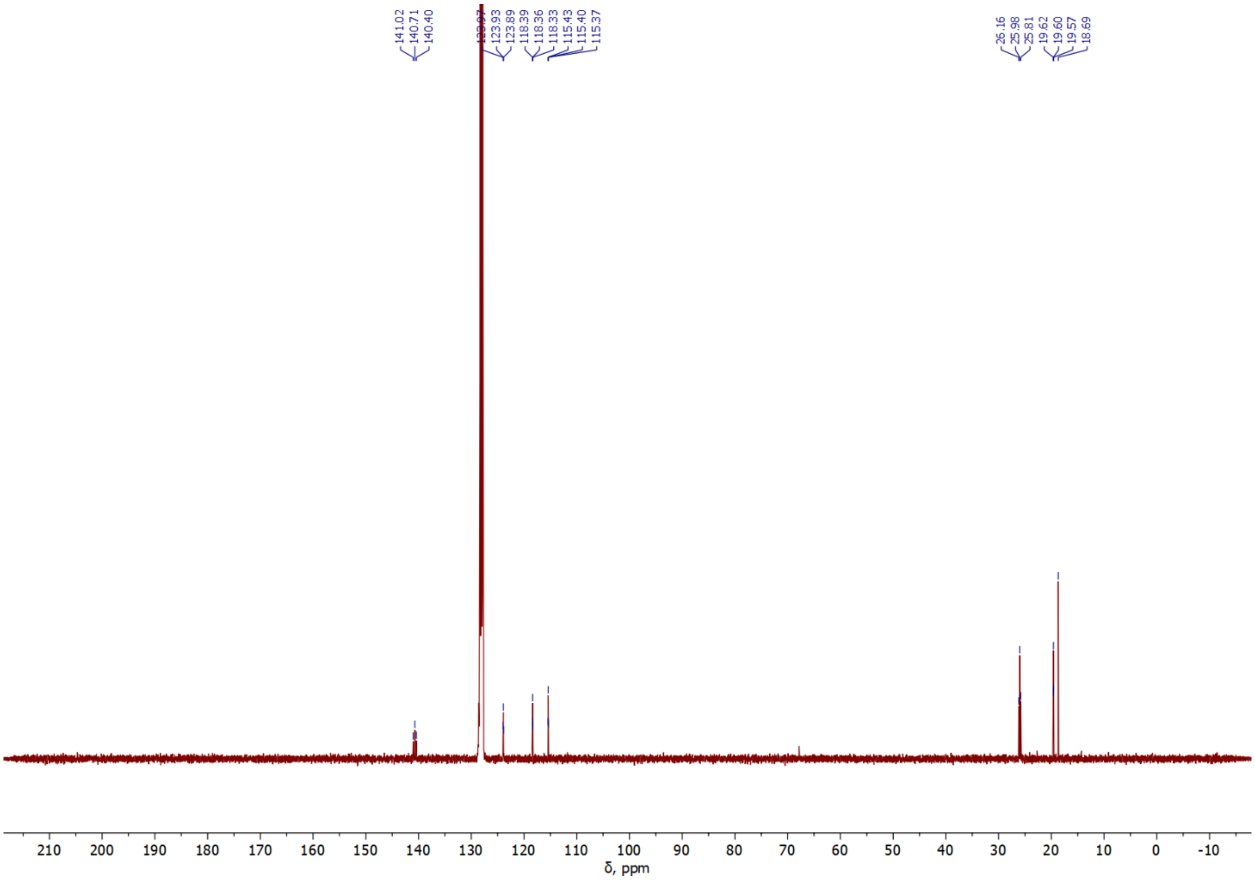

Figure S36. $^{13}C\{^{1}H\}$ NMR (101 MHz, C_6D_6) spectrum of **7** (98%, 2% of **4** not detected).

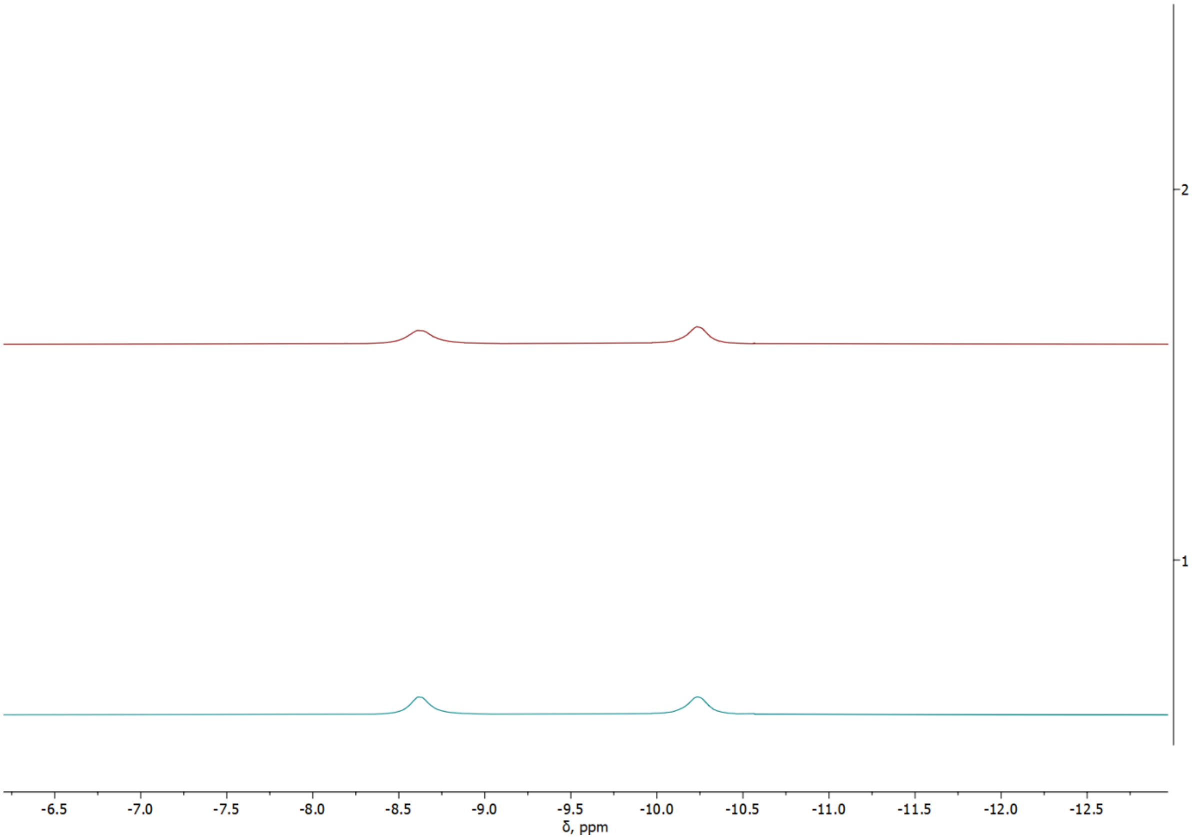

Figure S37. Comparison of ^{1}H NMR spectrum of **7** (Top) with $^{1}H\{^{11}B\}$ NMR spectrum of **7** (Bottom), (500 MHz, C_6D_6). Hydride region is shown, with 2% impurity of **4** excluded.

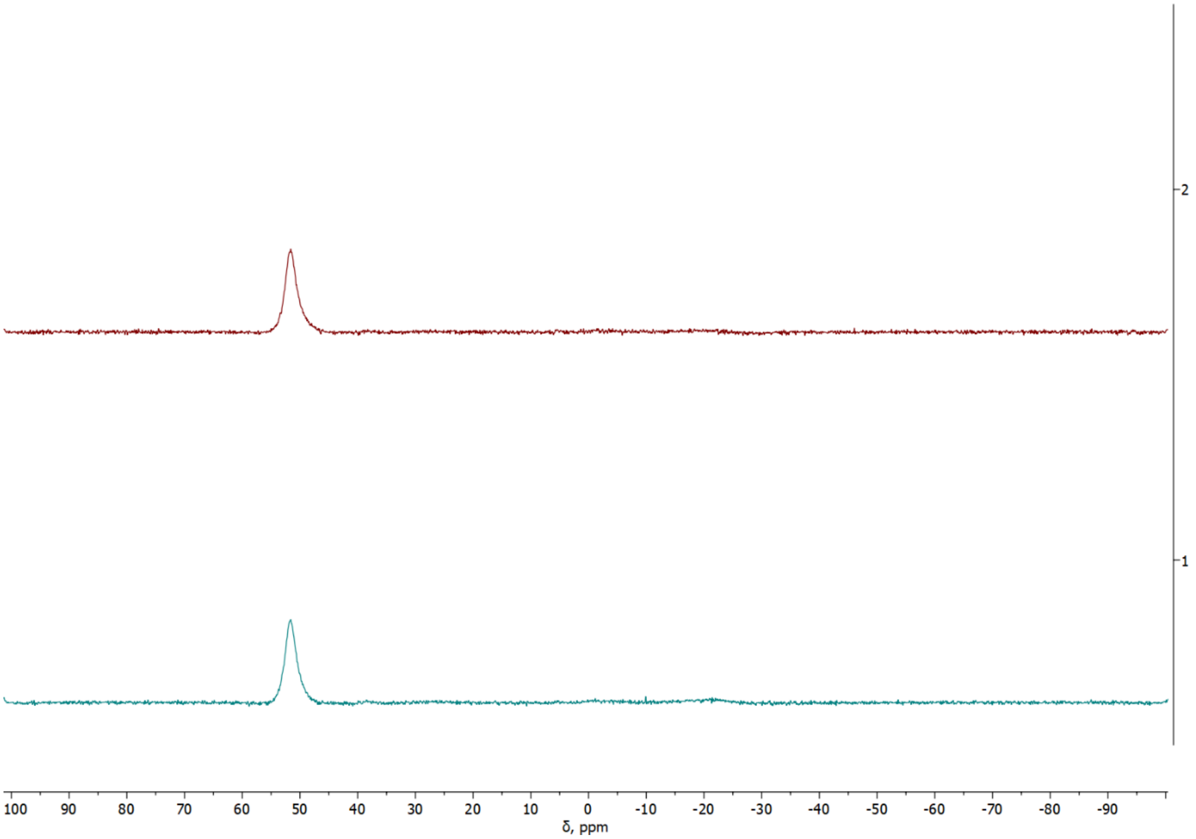

Figure S38. Comparison of $^{11}B\{^{1}H\}$ NMR spectrum of **7** (Top) with ^{11}B NMR spectrum of **7** (Bottom), (128 MHz, C_6D_6).

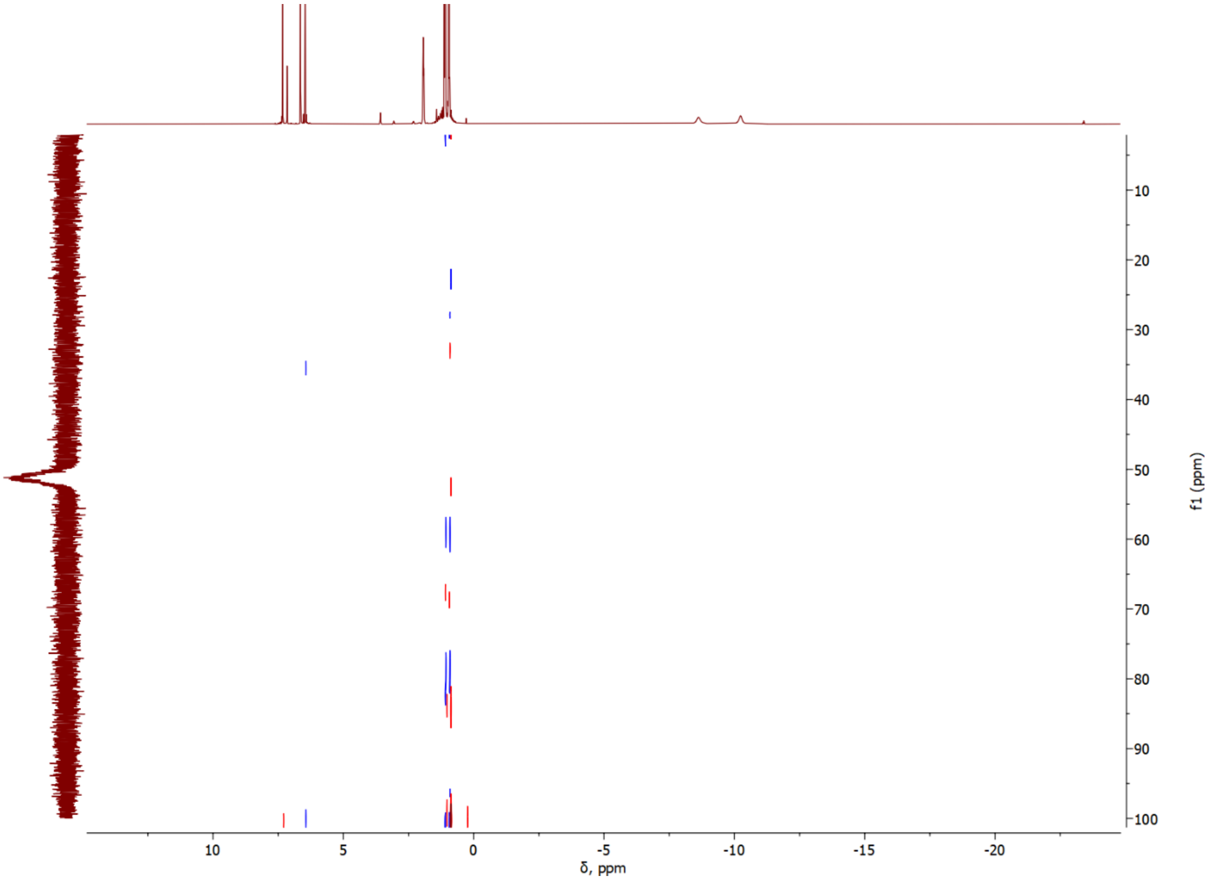

Figure S39. ^{1}H–^{11}B HMQC NMR spectrum for **7**.

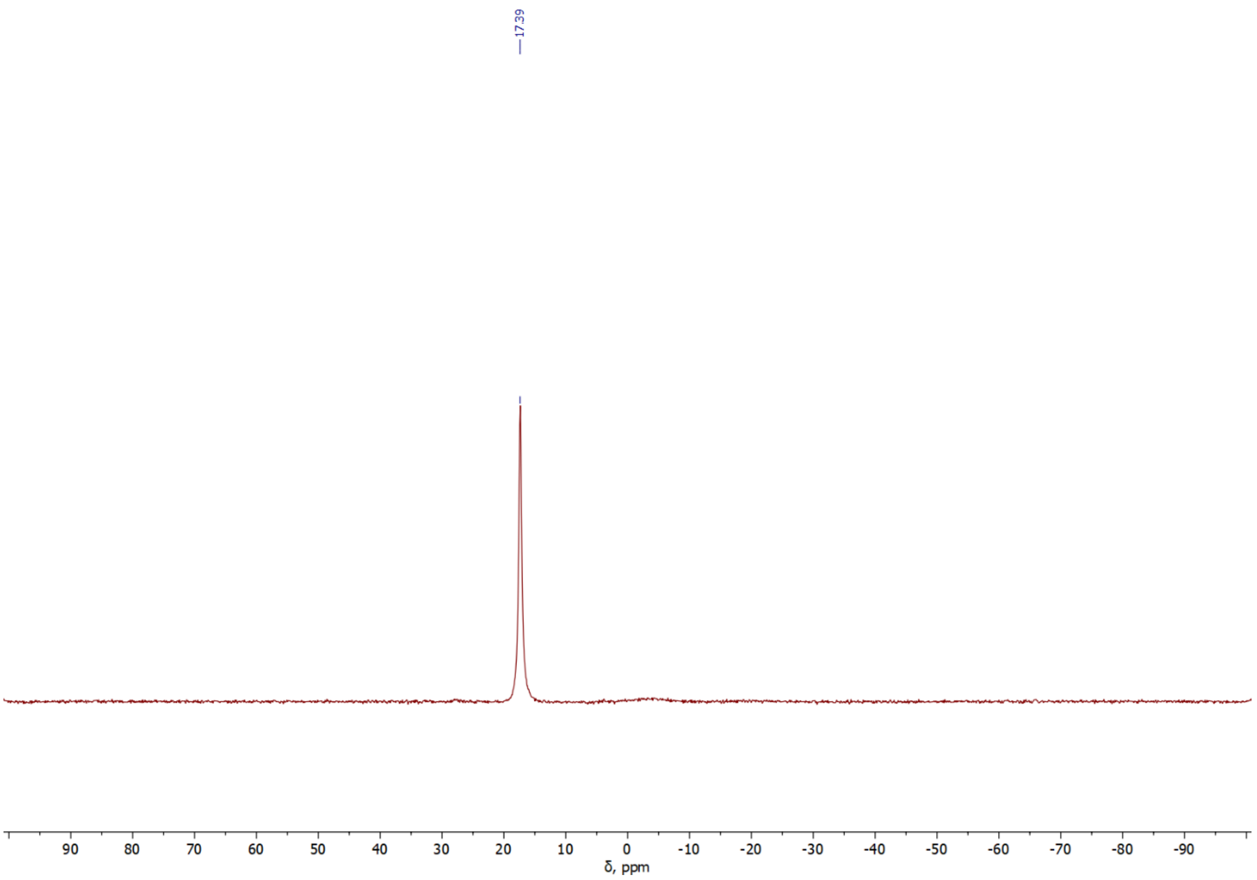

Figure S40. $^{11}B\{^{1}H\}$ NMR (128 MHz, THF/^{i}PrOH) of the reaction mixture aliquot for **8**.

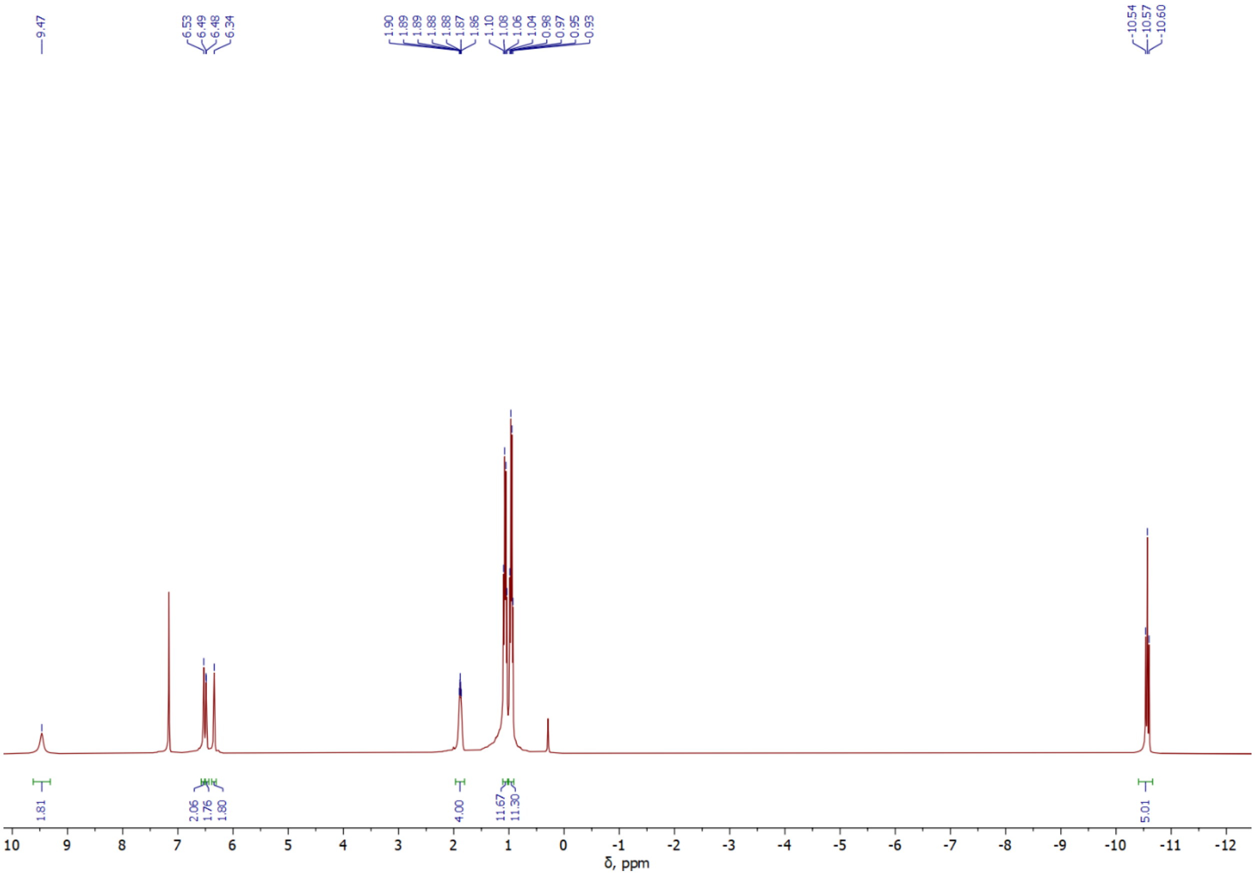

Figure S41. ^{1}H NMR (400 MHz, C_6D_6) of **8**, silicone grease present at δ 0.29 ppm.

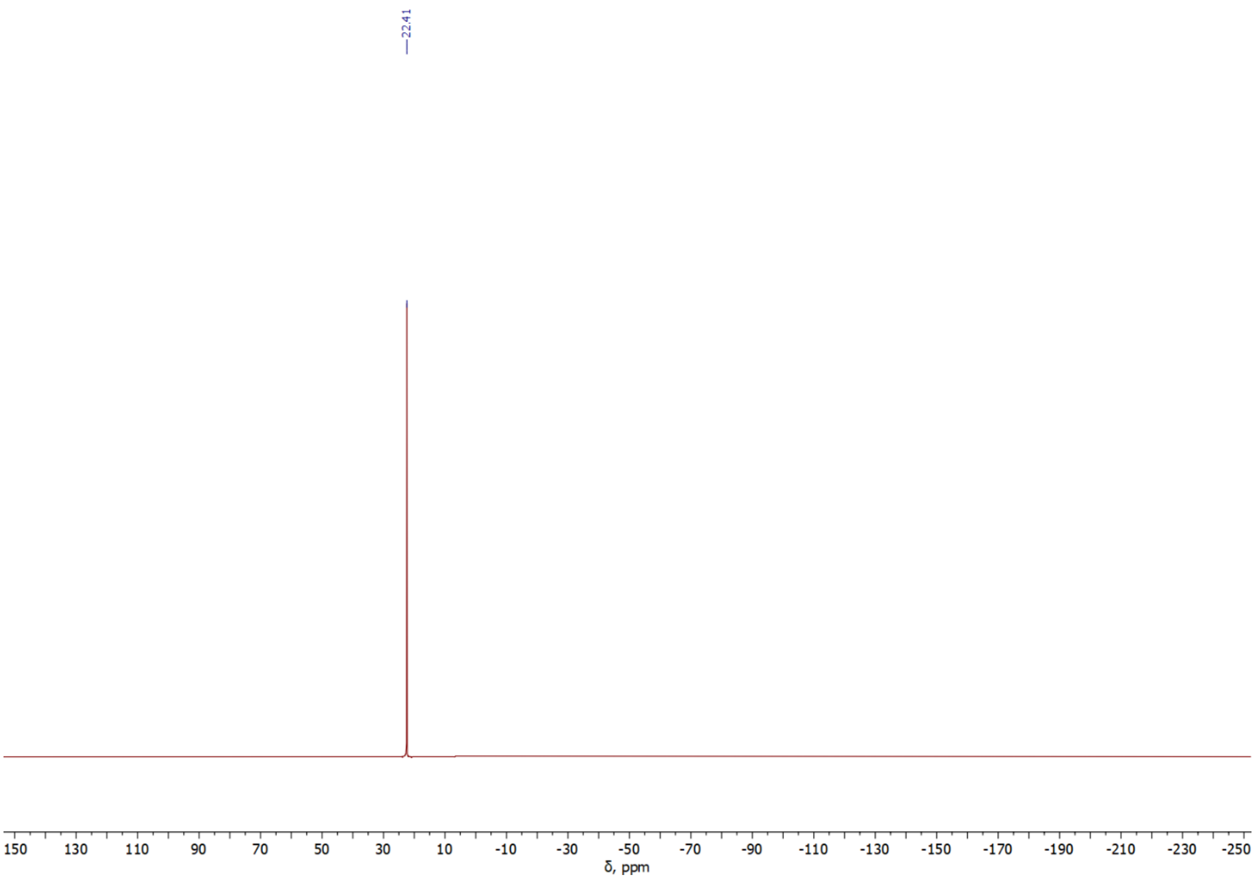

Figure S42. $^{31}P\{^{1}H\}$ NMR (162 MHz, C_6D_6) of **8**.

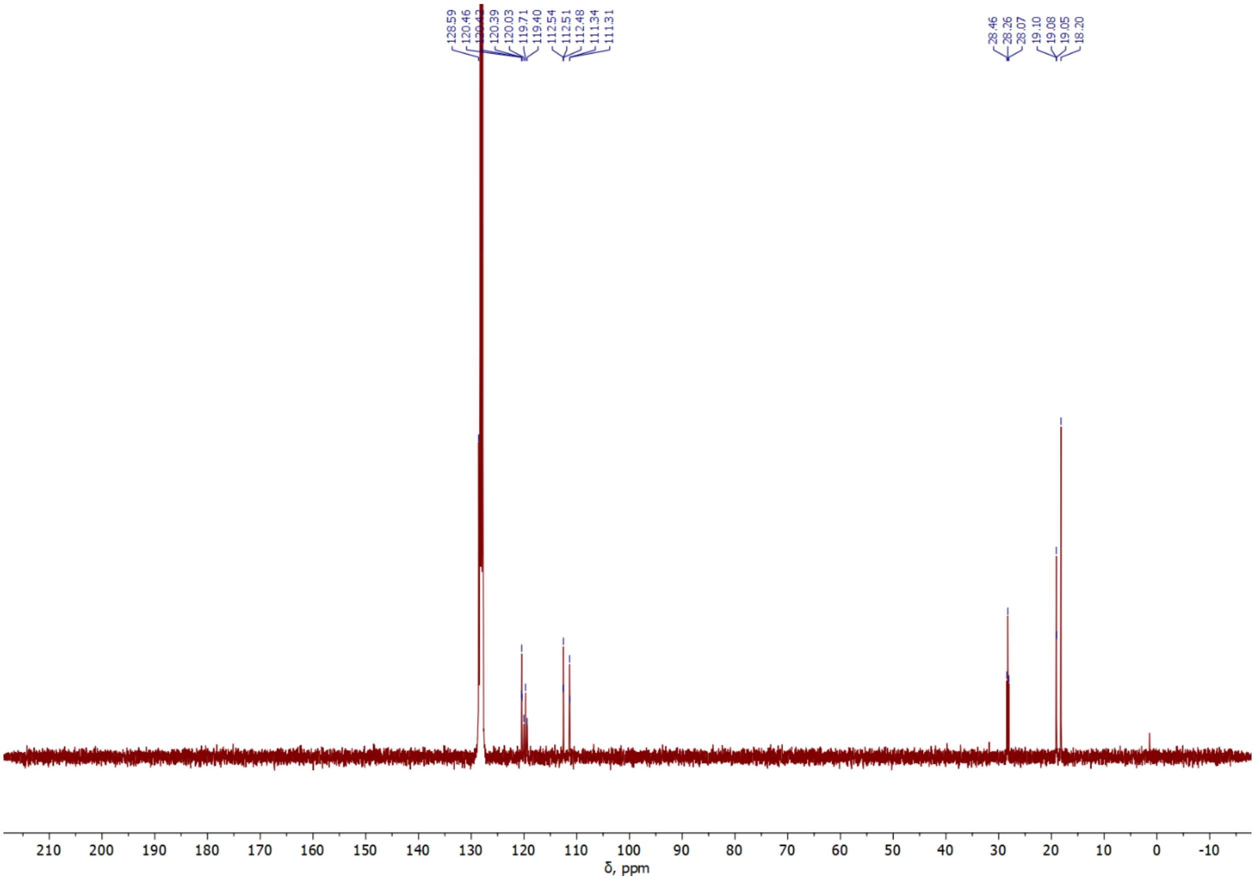

Figure S43. $^{13}C\{^{1}H\}$ NMR (101 MHz, C_6D_6) of **8**.

III. X-Ray Structural Analysis

X-Ray data collection, reduction, solution, and refinement for 6b (CCDC 2378572)

A Leica M80 microscope was used to identify a suitable single **orange plate-shaped** crystal of **6a** showing well defined faces with dimensions 0.07 × 0.05 × 0.01 mm^3 from a representative sample of crystals of the same habit. The crystal mounted on a nylon loop was then placed in a cold nitrogen stream (Oxford) maintained at T = 100.00(10) K.

Crystal screening, unit cell determination, and data collection were carried out using a XtaLAB Synergy, Dualflex, HyPix diffractometer. The diffraction pattern was indexed and the total number of runs and images was based on the strategy calculation from the program CrysAlisPro system.[1] Data were measured using ω scans with Cu K_α radiation. Data was collected to a maximum resolution of Θ= 74.830° (0.80 Å). The unit cell was refined using CrysAlisPro 1.171.43.98a[1] on 13185 reflections, 82 % of the observed reflections.

Integrated Intensity information for each reflection was obtained by reduction of data frames using CrysAlisPro 1.171.43.98a[1].The final completeness is 100.00 % out to 74.830° in Θ. A gaussian absorption correction was performed using CrysAlisPro 1.171.43.98a.[1] Numerical absorption correction based on gaussian integration over a multifaceted crystal model Empirical absorption correction using spherical harmonics, implemented in SCALE3 ABSPACK scaling algorithm. The absorption coefficient μ of this material is 25.010 mm^{-1} at this wavelength (λ = 1.54184Å) and the minimum and maximum transmissions are 0.551 and 0.939.

Systematic reflection conditions and statistical tests of the data suggested the space group $I2$ and

was confirmed by ShelXT[2] structure solution program using dual methods. The structure was refined by full matrix least squares minimisation on $\boldsymbol{F^2}$ using version 2019/1 of ShelXL 2019/1.[3] All non-hydrogen atoms were refined anisotropically. Hydrogen atom positions were calculated geometrically and refined using the riding model.

X-Ray data collection, reduction, solution, and refinement for 7 (CCDC 2378573)

A Leica M80 microscope was used to identify a suitable single **brown plate-shaped** crystal of **7** showing well defined faces with dimensions 0.31 × 0.09 × 0.02 mm^3 from a representative sample of crystals of the same habit. The crystal mounted on a nylon loop was then placed in a cold nitrogen stream (Oxford) maintained at T = 293(2) K.

Crystal screening, unit cell determination, and data collection were carried out using a XtaLAB Synergy, Single source at home/near, Eiger2 1M diffractometer. The diffraction pattern was indexed and the total number of runs and images was based on the strategy calculation from the program CrysAlisPro system (CCD 43.101a 64-bit (release 13-12-2023)).[1] Data were measured using ω scans with Ag K_α radiation. Data was collected to a maximum resolution of $\Theta = 25.558^\circ$ (0.65 Å). The unit cell was refined using CrysAlisPro 1.171.43.98a[1] on 71879 reflections, 68 % of the observed reflections.

Integrated Intensity information for each reflection was obtained by reduction of data frames using CrysAlisPro 1.171.43.98a.[1] The final completeness is 99.80 % out to 25.558° in Θ. A gaussian absorption correction was performed using CrysAlisPro 1.171.43.98a[1] Numerical absorption correction based on gaussian integration over a multifaceted crystal model Empirical absorption correction using spherical harmonics, implemented in SCALE3 ABSPACK scaling algorithm. The absorption coefficient μ of this material is 3.162 mm^{-1} at this wavelength (λ = 0.56087Å) and the

minimum and maximum transmissions are 0.664 and 1.000.

Systematic reflection conditions and statistical tests of the data suggested the space group $P2_1/n$ and was confirmed by ShelXT 2018/2[2] structure solution program using dual methods. The structure was refined by full matrix least squares minimisation on $\boldsymbol{F^2}$ using version 2019/1 of ShelXL 2019/1.[3] All non-hydrogen atoms were refined anisotropically. Hydrogen atom positions were calculated geometrically and refined using the riding model.

IV. SI References

(1) Rigaku Oxford Diffraction. *CrysAlisPro Software System*.
(2) Sheldrick, G. M. SHELXT – Integrated Space-Group and Crystal-Structure Determination. *Acta Cryst A* **2015**, *71*, 3–8.
(3) Sheldrick, G. M. Crystal Structure Refinement with SHELXL. *Acta Cryst C* **2015**, *71*, 3–8.
